# Outer membrane vesicles secreted by avian pathogenic *Escherichia coli* promote its survival within macrophages and systemic infection by inducing endoplasmic reticulum stress-mediated autophagy flux blockade

**DOI:** 10.1186/s13567-025-01679-6

**Published:** 2026-01-27

**Authors:** Tongtong Cui, Zhe Li, Jiayin Gao, Zhou Miao, Fayin Li, Ying Shao, Zhenyu Wang, Jiumeng Sun, Xiangjun Song, Kezong Qi, Jian Tu

**Affiliations:** 1 Anhui Province Key Laboratory of Veterinary Pathobiology and Disease Control, College of Veterinary Medicine, Anhui Agricultural University, Hefei, 230036 People’s Republic of China; 2 Anhui Province Engineering Laboratory for Animal Food Quality and Bio-Safety, College of Veterinary Medicine, Anhui Agricultural University, Hefei, 230036 People’s Republic of China; 3 Joint Research Center for Food Nutrition and Health of IHM, Hefei, 230036 People’s Republic of China

**Keywords:** Avian pathogenic *Escherichia coli*, outer membrane vesicles, macrophages, endoplasmic reticulum stress, incomplete autophagy, systemic infection, immune evasion

## Abstract

Avian pathogenic *Escherichia coli* (APEC) is an extraintestinal pathogenic *Escherichia coli* that primarily causes avian colibacillosis, leading to localized or systemic infections. Its persistent survival within host macrophages is a critical component of the systemic infection process. In this process, outer membrane vesicles (OMVs) secreted by APEC play an important role; however, the interactions between OMVs and macrophages and their effects on bacterial intracellular survival remain unclear. This study demonstrated that APEC-derived OMVs induced intracellular Ca^2+^ release and reactive oxygen species (ROS) accumulation in macrophages, thereby activating all three branches of the protein kinase RNA-like ER kinase (PERK), inositol-requiring enzyme 1 (IRE1), and activating transcription factor 6 (ATF6) pathways of the unfolded protein response (UPR), leading to sustained endoplasmic reticulum stress (ERS). Additionally, OMVs disrupted the acidic environment of lysosomes, inhibiting autophagosome–lysosome fusion and leading to abnormal accumulation of autophagy marker proteins LC3-II and p62, resulting in ERS-mediated autophagy flux blockade (incomplete autophagy). Importantly, this ERS-dependent autophagy dysfunction significantly impaired the ability of macrophages to clear pathogens, thereby promoting intracellular proliferation and survival of APEC. Intervention with ERS inhibitors effectively alleviated ERS and restored autophagosome–lysosome fusion, significantly reducing APEC intracellular survival within macrophages (*p* < 0.01) and bacterial loads in chick tissues (trachea: *p* < 0.001, lungs: *p* < 0.001, liver: *p* < 0.05, spleen: *p* < 0.001). These results indicated that APEC triggers ERS in host macrophages by secreting OMVs, thereby causing autophagy flux blockade, escaping host immune clearance, achieving sustained intracellular survival, and ultimately leading to systemic infection. This study provides new insights into the role of OMVs in regulating the innate immune response of macrophages during APEC infection.

**Supplementary Information:**

The online version contains supplementary material available at 10.1186/s13567-025-01679-6.

## Introduction

Avian pathogenic *Escherichia coli* (APEC) is one of the primary pathogens responsible for severe systemic infections in poultry, leading to conditions such as septicemia, airsacculitis, pericarditis, and pneumonia, collectively referred to as avian colibacillosis, which causes substantial economic losses to the poultry industry [1]. APEC infection typically begins with colonization of the respiratory tract mucosa, followed by breach of the epithelial barrier and invasion of the bloodstream, leading to systemic dissemination affecting multiple organs such as the liver and pericardium. This ultimately results in fibrinous suppurative lesions and bacteremia, causing high mortality rates [2]. Although the pathogenicity of APEC is well established, the specific molecular mechanisms underlying its ability to breach the respiratory tract barrier and achieve systemic dissemination remain incompletely understood. Previous studies have indicated that early innate immune responses, particularly those of local macrophages and heterophils, play a critical role in limiting APEC colonization and disease progression [3]. Notably, APEC can persist long-term within macrophages, a capability believed to be a key mechanism for evading immune clearance, facilitating systemic dissemination, and ultimately causing host mortality [4]. Therefore, elucidating the survival strategies of APEC within macrophages is of great significance for understanding the process by which local infection progresses to a lethal systemic disease. Although various APEC virulence factors have been identified, the molecular mechanisms underlying their proliferation and survival within macrophages remain unclear.

During colonization, pathogens are exposed to multiple environmental stresses from the host. Outer membrane vesicles (OMVs) serve as an independent bacterial stress response mechanism and are significantly activated during this process. OMVs are rich in virulence factors, toxins, and other bioactive proteins [5]. Increasing evidence suggests that pathogens utilize OMVs as an efficient delivery system to co-deliver or directly transport effector molecules (including active toxins) into the host cell cytoplasm, thereby manipulating host signal transduction, disrupting cellular functions, and promoting bacterial survival and dissemination [6]. For example, the OMVs of *Neisseria gonorrhoeae* can induce mitophagy, inhibit reactive oxygen species (ROS) production, and enhance intracellular survival [7]. *Salmonella* upregulates OMV production through the PhoP–PhoQ system, resists complement killing in a dose-dependent manner, and promotes the release of virulence factors such as PagC, facilitating local and systemic bacterial dissemination [8]. Recent studies have shown that macrophages, upon recognizing pathogens and their secreted factors, can activate multiple programmed cell death pathways to limit microbial replication and enhance immune clearance. However, there is also evidence that, in certain infection scenarios, macrophage death responses may aggravate rather than control infection [9, 10]. The latest studies have shown that OMVs secreted by APEC can induce necroptosis in chicken macrophage cells through a receptor-interacting serine/threonine-protein kinase 1 (RIPK1)-dependent pathway [11]. Given that APEC has a significant ability to survive for a long time within macrophages, this finding suggests that its OMVs may contribute to bacterial persistence and spread by regulating host innate immune signaling or impairing immune cell function. However, direct evidence to clarify the immune regulatory functions of APEC OMVs and their specific roles in systemic infections remains lacking.

Pathogen invasion typically triggers two typical stress responses in host cells: autophagy and endoplasmic reticulum stress (ERS) or the unfolded protein response (UPR) [12]. The endoplasmic reticulum (ER) is a key site for protein synthesis and processing, and its homeostasis is critical for cell survival. Disruption of ER function by viral, fungal, parasitic, or bacterial infections leads to the accumulation of unfolded or misfolded proteins, thereby inducing ERS and activating the UPR. The UPR is mediated by three classic signaling pathways: the protein kinase RNA-like ER kinase (PERK), the inositol-requiring enzyme 1 (IRE1), and the activating transcription factor 6 (ATF6). These pathways induce the expression of key factors such as lucose-regulated protein 78 (GRP78)/binding immunoglobulin protein (BiP) and C/EBP homologous protein (CHOP), and restore ER homeostasis by promoting protein degradation and enhancing autophagic activity [13, 14]. Increasing evidence suggests that various pathogens can exploit or hijack UPR signaling to shape an intracellular environment favorable for their replication. For example, *Brucella* achieves intracellular replication through host UPR and ER-derived vesicles [15]. UPR activation is often accompanied by increased autophagy activity, which is crucial for maintaining ER homeostasis [16]. However, if ERS persists without resolution, UPR may also shift toward inducing programmed cell death, with outcomes that can either limit or potentially exacerbate infection. For example, the subtilase cytotoxin (SubAB) toxin secreted by Shiga toxin-producing *Escherichia coli* activates the UPR by cleaving the BiP protein, thereby inhibiting NLRP3 inflammasome assembly or promoting host cell apoptosis [17, 18]. These findings indicate that ERS/UPR not only centrally regulates cell homeostasis but also broadly participates in innate immune responses. However, its specific regulatory mechanisms in the context of infection remain to be further elucidated.

Autophagy is a key mechanism of the host innate immune defense, limiting infection by targeting intracellular pathogens to lysosomes for degradation. However, some pathogens have evolved strategies to evade or even exploit the autophagy process to maintain their survival and replication within cells [19]; For example, *Helicobacter pylori*, *Borrelia burgdorferi*, and uropathogenic *Escherichia coli* can block autophagosome acidification or lysosomal degradation, thereby impairing autophagosome maturation and achieving immune evasion [20]. Research also suggests that the ER plays a crucial role in phagosome formation: the absence of ER-resident proteins such as calreticulin and calnexin weakens phagocytic activity [21]. Although multiple lines of evidence support the involvement of the ER in phagosome biogenesis [22], the “ER–phagosome model” remains controversial. Notably, there may be cross-regulatory networks between ER function and autophagy degradation pathways, and their roles in infection processes remain to be systematically elucidated.

This study evaluated the role of APEC-derived OMVs in respiratory tract colonization, systemic dissemination, and survival within HD11 macrophages through in vivo and in vitro experiments. Furthermore, we investigated whether OMVs secreted by APEC activate UPR to induce ERS and analyzed whether ERS mediates OMV-induced alterations in autophagy flux, thereby promoting APEC escape from macrophage-mediated immune clearance. Collectively, we reveal a mechanism whereby OMVs promote APEC survival within macrophages and systemic infection by inducing ERS-mediated autophagy flux blockade.

## Materials and methods

### Ethics statement

All animal experiments were conducted by the Anhui Province Laboratory Animal Management Measures and approved by the Animal Experiment Ethics Committee of Anhui Agricultural University (KJLLXM2025104). This study established clear humane endpoints for the laboratory animals. When animals exhibited significant weight loss (≥ 10%), persistent reduction in food intake, decreased mobility, lethargy, or other signs of unrelievable distress, the experiment was immediately terminated. Euthanasia was performed in accordance with ethical guidelines to minimize animal suffering.

### Strains and plasmid

The clinical strain AE81 (serotype O166:H45) used in this study was isolated from the lung tissue of a chicken that had died of *Escherichia coli* and exhibiting symptoms of sepsis, collected from Anhui Province, China [23]. The OMV secretion-deficient strain AE81Δ*ypjA* [11], and the mCherry-GFP-LC3 plasmid are stored at the Anhui Provincial Key Laboratory of Veterinary Pathology and Epidemic Prevention (Anhui, China). AE81 and AE81Δ*ypjA* strains were cultured at 37 °C in standard Luria–Bertani (LB) broth or LB agar medium without antibiotics. Bacterial cultures in broth were grown with shaking at 200 rpm to the logarithmic phase (OD_600_ ≈ 0.6–0.8, approximately 3–5 h). Agar plates were used for colony isolation and viable cell counts.

### Reagents and antibodies

The antibodies and chemicals used in this study are detailed in Table 1.

**Table 1:** **Antibodies and chemical reagents used in this study**

Antibody or chemical reagent	Source	Dilution
Mouse anti-β-actin monoclonal antibody	Proteintech, Hubei, China	1:20 000
Mouse anti-OmpA polyclonal antibody	Anhui Provincial Key Laboratory of Veterinary Pathology and Disease Prevention and Control, Anhui, China	1:500
Rabbit anti-OmpF polyclonal antibody	Biorbyt, Cambridge, UK	1:500
Rabbit anti-GRP78/BiP polyclonal antibody	Abmart, Shanghai, China	1:5000
Rabbit anti-PERK polyclonal antibody	Abmart, Shanghai, China	1:5000
Rabbit anti-p-PERK polyclonal antibody	Abmart, Shanghai, China	1:5000
Rabbit anti-elF2α polyclonal antibody	Proteintech, Hubei, China	1:2000
Rabbit anti-p-elF2α polyclonal antibody	Proteintech, Hubei, China	1:2000
Rabbit anti-CHOP polyclonal antibody	Abmart, Shanghai, China	1:5000
Rabbit anti-IRE1 polyclonal antibody	Abmart, Shanghai, China	1:5000
Rabbit anti-p-IRE1 polyclonal antibody	Abmart, Shanghai, China	1:5000
Rabbit anti-ATF6 polyclonal antibody	Boster, Hubei, China	1:2000
Rabbit anti-LC3B polyclonal antibody	Proteintech, Hubei, China	1:3000
Rabbit anti-p62/SQSTM1 polyclonal antibody	Proteintech, Hubei, China	1:20 000
Rabbit anti-Beclin1 polyclonal antibody	Bioss, Beijing, China	1:2000
Mouse anti-LAMP1 monoclonal antibody	Zenbio, Sichuan, China	1:300
Goat anti-rabbit horseradish peroxidase-labeled antibody	Zenbio, Sichuan, China	1:1000
Goat anti-mouse horseradish peroxidase-labeled antibody	Zenbio, Sichuan, China	1:1000
FITC-labeled goat anti-rabbit IgG (H + L)	Beyotime, Shanghai, China	1:500
Cy3-labeled goat anti-mouse IgG (H + L)	Beyotime, Shanghai, China	1:500
4-Phenylbutyric acid (4-PBA)	MedChemExpress, Shanghai, China	2 mM
CQ	MedChemExpress, Shanghai, China	10 μM
Mito-TEMPO	MedChemExpress, Shanghai, China	10 μM
Bafilomycin A1 (BafA1)	MedChemExpress, Shanghai, China	100 nM

### Isolation and purification of APEC OMVs

OMVs were extracted from the supernatant of AE81 culture using a modified protocol described by Welsh et al. [24]. Firstly, the AE81 strain was cultured in LB broth medium until OD_600_ = 1. The supernatant was collected and filtered successively with 0.45-μm and 0.22-μm filters. Subsequently, the filtrate was concentrated using 100-kDa ultrafiltration tubes (Millipore, MA, USA), and then centrifuged at 4 °C with a relative centrifugal force of 39 000 × *g* for 3 h for crude extraction. The OMV precipitate was resuspended in filtered 10 mM 4-(2-hydroxyethyl)-1-piperazineethanesulfonic acid (HEPES) buffer (pH 7.4) containing 45% (w/v) OptiPrep (Sigma-Aldrich, MO, USA), 0.85% NaCl. To further purify the OMVs, the crude OMVs extract was added onto a 40% (bottom) and 25% (top) gradient OptiPrep solution and centrifuged at 298 700 × *g* for 6 h at 4 °C. After centrifugation, the fraction corresponding to the 28–31% OptiPrep layer was collected, diluted eightfold with sterile HEPES buffer, and centrifuged again at 39 000 × *g* for 3 h at 4 °C to remove residual OptiPrep. Finally, the precipitate on the centrifuge tube was suspended in sterile phosphate-buffered saline (PBS) to obtain the purified OMVs, which was stored at −80 ℃.

The protein concentration of OMVs was determined using the bicinchoninic acid (BCA) Protein Concentration Assay Kit (Sparkjade, Shandong, China). When OMVs were visualized by a laser scanning confocal microscopy (Leica, Wetzlar, Germany), DiO dye (10 μM) (Yeasen, Shanghai, China) was used for labeling, and a dye-only control treated with the same purification process was included.

### Cell culture

The HD11 chicken macrophage-like cell line used in this study was maintained by the Anhui Provincial Key Laboratory of Veterinary Pathology and Epidemic Prevention. HD11 cells were cultured in Dulbecco’s modified Eagle’s medium (DMEM; VivaCell, Shanghai, China) supplemented with 10% fetal bovine serum (ExCell Bio, Shanghai, China) and 1% antibiotics (100 U/mL penicillin and 100 μg/mL streptomycin) at 37 °C in a humidified atmosphere containing 5% CO_2_. Transfection of HD11 cells with plasmid DNA (mCherry-GFP-LC3 plasmid) was performed using Lipofectamine 2000 (Thermo Fisher, Massachusetts, USA) and Opti-MEM medium (Gibco, California, USA). For OMV treatment and bacterial infection experiments, HD11 cells were seeded at 2 × 10^5^ cells/well in 24-well plates and cultured for 16 h. The medium was then removed, and cells were incubated with DMEM-diluted OMVs resuspension or bacterial suspension (OD_600_ = 1). For experiments requiring laser confocal microscopy, 1 × 10^5^ cells were seeded in 24-well plates with round glass coverslips (NEST, Jiangsu, China).

### Transmission electron microscopy

OMVs stored at −80 °C were thawed and thoroughly mixed before being applied onto copper grids. After staining with 2% phosphotungstic acid and air-drying, the morphology of the OMVs was observed using a transmission electron microscope (HITACHI, Tokyo, Japan).

HD11 cells treated with APEC, APECΔ*ypjA*, or OMVs were fixed overnight with 2.5% glutaraldehyde, post-fixed with 1% osmium tetroxide for 1–2 h, dehydrated through a graded ethanol series, embedded in epoxy resin, and observed using a transmission electron microscope to obtain images of the endoplasmic reticulum and autophagosomes.

### Nanoparticle tracking analysis

To determine the concentration and size distribution of OMVs, a ZetaView PMX 110 nanoparticle tracking analysis system (Particle Metrix, Germany) was used. Measurements were performed using a 405-nm laser to assess particle dispersion and concentration. Prior to measurement, OMVs samples were diluted with PBS to obtain a final concentration in the range of 1 × 10^7^ to 1 × 10^9^ particles/mL. The diluted samples were then analyzed to determine the particle size distribution and concentration of OMVs.

### RNA extraction and quantitative real-time PCR (qPCR)

Total RNA was extracted from cells using the SPARKeasy Tissue/Cell RNA Rapid Extraction Kit (Sparkjade, Shandong, China). The extracted RNA was reverse-transcribed into complementary DNA (cDNA) using the SPARKscript II RT Plus Kit (With gDNA Eraser) (Sparkjade, Shandong, China).

Using *β-actin* as an internal control (whose expression level remains relatively stable under ERS conditions and is not significantly affected by UPR signaling pathway activation), qPCR was employed to detect the relative transcription levels of ERS-related gene *GRP78/BiP*, PERK pathway-related genes *ATF4* and *CHOP*, IRE1 pathway-related genes *EDEM1* and *ERdj4*, and the *ATF6* gene. The primer sequences are listed in Table 2. The total volume of the qPCR reaction was 20 μL, containing 1 μL of cDNA, 1 μL of forward and reverse primers, 7 μL of ddH₂O, and 10 μL of SYBR Green Master Mix (No Rox) (Yeasen, Shanghai, China). Each sample was replicated three times, and relative gene expression levels were calculated using the 2^−ΔΔCt^ method.

**Table 2:** **Primers of the detected genes**

Gene	Sequence (5′–3′)	Amplicon size
*β-actin*-F *β-actin*-R	CCGCTCTATGAAGGCTACGC CTCTCGGCTGTGGTGGTGAA	1128 bp
*GRP78/BiP*-F *GRP78/BiP*-R	GAATCGGCTAACACCAGAGGA CGCATAGCTCTCCAGCTCATT	1959 bp
*CHOP*-F *CHOP*-R	GCATCCAGAAGGAAGAGCGT GGGGACGTTGAGACAGCAAT	468 bp
*ATF4*-F *ATF4*-R	TGGAACATCTGGAAGCCACC CCTTTCACCTTTGCTGACGC	1065 bp
*ATF6*-F *ATF6*-R	CGTCGTCTGAACCACTTACTGA CCTTCTTTCCTAACAGCCACAC	2016 bp
*EDEM1*-F *EDEM1*-R	CTTCGGCTACGACAGCTACA GGCTTCCCAAAACCCTGATG	1851 bp
*ERdj4*-F *ERdj4*-R	TGACCGCCAGATCAAGAAGG GCCCGGAACAGTCTGTGTAT	651 bp

### Western blot analysis

HD11 cells were lysed using radioimmunoprecipitation assay (RIPA) lysis buffer (Epizyme Biotech, Shanghai, China) supplemented with a mixture of protease and phosphatase inhibitors (Epizyme Biotech, Shanghai, China), and the lysate was collected. The supernatant obtained after centrifugation of the lysate served as the total protein sample. Omni-Easy™ Instant Protein Loading Buffer (Epizyme Biotech, Shanghai, China) was added to the protein samples, which were then denatured by heating at 100 °C for 10 min. Approximately 30 μg of protein sample was separated by electrophoresis on a 10% or 12.5% sodium dodecyl sulfate (SDS)-polyacrylamide gel electrophoresis (PAGE) gel and transferred to polyvinylidene fluoride (PVDF) membranes (Millipore, Massachusetts, USA). The membranes were then blocked with 5% nonfat dry milk at 37 °C for 2 h and incubated with the primary antibody at 4 °C for 12–18 h. The next day, the membranes were washed three times with Tris-buffered saline with Tween-20 (TBST) buffer (Servicebio, Hubei, China) for 10 min each. They were then incubated with the secondary antibody at 37 °C for 1 h, followed by washing three times with TBST (10 min each). Finally, ultrasensitive enhanced chemiluminescence (ECL) reagents (Epizyme Biotech, Shanghai, China) were used and the target bands were displayed through the chemiluminescence imaging system. Quantitative analysis of band grayscale values was performed using ImageJ software. β-Actin was used as the internal reference protein to calculate the relative expression levels of the target proteins.

### Cell viability assay

The viability of cells was determined by using the Cell Counting Kit-8 (TargetMOI, USA). Cells (2 × 10^4^) were seeded in a 96-well plate and cultured overnight. The experiment included the following groups: blank group (wells without cells), control group (untreated cells), OMV-treated group (treated with 200 μg/mL OMVs for 12 h), and OMVs + ERS inhibitor group (treated in the presence of 2 mM 4-PBA for 12 h). After treatment, culture medium was removed and 100 μL of cell culture medium containing 1/10 volume of Cell Counting Kit-8 was added, followed by incubation at 37 °C for 40 min. The absorbance was measured at a wavelength of 450 nm using a multimode microplate reader (Molecular Devices, CA, USA). Each experimental group had three replicate wells and was performed independently three times. Cell viability was calculated using the following formula:

$$ {\text{Cell viability}}\left( {{\% }} \right) = \frac{{\left( {{\mathrm{OD}}450,{\mathrm{experimental}} - {\mathrm{OD}}450,{\mathrm{blank}}} \right)}}{{\left( {{\mathrm{OD}}450,{\mathrm{control}} - {\mathrm{OD}}450,{\mathrm{blank}}} \right)}}\, \times \,100\%. $$

The results are expressed as percentages representing relative cell viability.

### ROS measurements

The intracellular ROS level was detected using 2′,7′-Dichlorodihydrofluorescein diacetate (DCFH-DA; Solarbio, Beijing, China). HD11 cells were treated with APEC, APECΔ*ypjA*, or OMVs, washed twice with PBS, and then incubated with DCFH-DA diluted 1:5000 in serum-free DMEM in a 37 °C cell culture incubator for 30 min. The cells were then washed three times with DMEM and observed using an inverted fluorescence microscope (Olympus, Tokyo, Japan). Rosup reagent (Solarbio, Beijing, China) was used as a positive control. Quantitative analysis of fluorescence images was conducted using ImageJ software.

### Ca^2+^ measurements

The intracellular Ca^2+^ concentration was detected using the fluorescent calcium indicator Fluo-3-AM (Solarbio, Beijing, China). HD11 cells treated with APEC, APECΔ*ypjA*, or OMVs were washed three times with Hank’s balanced salt solution (HBSS) solution (Solarbio, Beijing, China) to remove residual medium. Cells were then incubated at 37 °C with 5 μM Fluo-3 AM working solution (diluted in HBSS) for 30 min to load the fluorescent probe. After incubation, the dye working solution was discarded, and cells were washed three times with HEPES buffer saline (Solarbio, Beijing, China) to remove unbound probe. To promote probe dealkylation, cells were further incubated at 37 °C in a cell culture incubator for 30 min in HBSS containing 1% fetal bovine serum. Following treatment, images were acquired using an inverted fluorescence microscope. Quantitative analysis of fluorescence images was conducted using ImageJ software.

### Immunofluorescence analysis

After treating HD11 cells with OMVs for 6 h, the cells were washed three times with PBS, fixed with 4% paraformaldehyde at room temperature for 15 min, washed three times with PBS, permeabilized with 0.3% Triton X-100 at room temperature for 15 min, and washed three times with PBS. The cells were blocked by incubating with PBS containing 5% fetal bovine serum for 2 h, then incubated with primary antibodies LC3 and lysosome-associated membrane protein 1 (LAMP1) overnight at 4 °C. After washing three times with TBST (10 min each), the cells were incubated with fluorescein isothiocyanate (FITC)-labeled goat anti-rabbit immunoglobulin G (IgG) (H + L) and Cy3-labeled goat anti-mouse IgG (H + L) at 37 °C for 1 h and washed three times with TBST (10 min each). Nuclear DNA was stained with 4′,6-diamidino-2-phenylindole (DAPI; Sparkjade, Shandong, China). Images were acquired using a laser confocal microscope. Under each condition, at least 40 cells were randomly selected from three independent experiments for blinded analysis. Fluorescence intensity was quantified using ImageJ software, Pearson’s correlation coefficient (*R*) was calculated using Coloc2 in ImageJ software with default settings, and colocalization scatter plots of LC3 and LAMP1 were generated using GraphPad Prism 8.

### ER staining analysis

After treatment of HD11 cells with DiO-OMVs for 6 h, the cells were washed three times with PBS and incubated with ER-Tracker (Beyotime, Shanghai, China) diluted at a ratio of 1:1000 in a 37 °C cell culture incubator for 30 min. Then, the cells were fixed with 4% paraformaldehyde at room temperature for 15 min and washed three times with PBS. Nuclear DNA was labeled with DAPI. The fluorescence localization of DiO-OMVs and ER-Tracker was observed using a laser confocal microscope, and images were obtained. Under each condition, at least 40 cells were randomly selected from three independent experiments for blinded analysis. Fluorescence intensity was quantified using ImageJ software, Pearson’s correlation coefficient (*R*) was calculated using Coloc2 in ImageJ software with default settings, and colocalization scatter plots of DiO and ER-Tracker were generated using GraphPad Prism 8.

### mCherry-GFP-LC3 assay

HD11 cells were transfected with the mCherry GFP-LC3 tandem fluorescent plasmid and cultured at 37 °C and 5% CO_2_ for 24 h to ensure full expression of fluorescent proteins. This fluorescent plasmid consists of an acid-sensitive green fluorescent protein (GFP) and an acid-insensitive red fluorescent protein (mCherry). Under neutral conditions, both GFP and mCherry emit fluorescence. However, in acidic environments, GFP fluorescence is quenched while mCherry continues to fluoresce. This system can be used to distinguish autophagosomes from autophagolysosomes, thereby reflecting the acidification status of lysosomes.

HD11 cells following transfection were treated with OMVs for 6 h with or without 4-PBA (2 mM) or Mito-TEMPO (10 μM). Lysosomal acid inhibitors chloroquine (CQ, 10 μM) and BafA1 (100 nM) were used as controls. Following treatment, cells were fixed with 4% paraformaldehyde at room temperature for 15 min and washed three times with PBS. Nuclear DNA was stained with DAPI. Fluorescent images of mCherry and GFP were acquired using a laser confocal microscope. Yellow puncta (GFP^+^mCherry^+^) in the images represent autophagosomes, while red puncta (mCherry^+^GFP^−^) represent autophagolysosomes. Under each condition, at least 40 cells were randomly selected from three independent experiments for blinded analysis. Fluorescence intensity was quantified using ImageJ software, Pearson’s correlation coefficient (*R*) was calculated using Coloc2 in ImageJ software with default settings, and colocalization scatter plots of mCherry and GFP were generated using GraphPad Prism 8.

### Lysosome staining analysis

Two lysosome-specific fluorescent probes were used for staining analysis: LysoTracker (Beyotime, Shanghai, China), which specifically labels lysosomal structures, and LysoSensor (Yeasen, Shanghai, China), which binds to acidic organelles via protonation and emits stronger fluorescence signals as the pH decreases. HD11 cells were treated with OMVs for 6 h with or without 4-PBA (2 mM). CQ (10 μM) was used as a control. The cells were incubated with 1:15 000 diluted LysoTracker and 1:1000 diluted LysoSensor at 37 °C in a cell culture incubator for 30 min, and washed three times with PBS. The cells were then fixed with 4% paraformaldehyde at room temperature for 15 min and washed three times with PBS. Nuclear DNA was labeled with DAPI, and the fluorescence intensity of LysoTracker and LysoSensor was detected and images were obtained using a laser confocal microscope. Each set of fluorescence imaging was performed under identical parameters with three independent replicates. Fluorescence intensity was quantified using ImageJ software, Pearson’s correlation coefficient (*R*) was calculated using Coloc2 in ImageJ software with default settings, and colocalization scatter plots for LysoTracker and LysoSensor were generated using GraphPad Prism 8.

### Bacterial infection analysis

HD11 cells were infected with APEC or APECΔ*ypjA* suspended in cell culture medium at OD_600_ = 1 at multiplicity of infection (MOI) of 100:1 for 2 h in the presence or absence of 4-PBA (2 mM), with three replicate wells. After infection, the bacterial suspension was discarded, and the cells were washed three times with PBS, followed by incubation in fresh medium containing 100 μg/mL gentamicin to kill extracellular bacteria.

At the indicated time points (1, 2, and 3 h post-treatment), the cells were washed three times with PBS and lysed with 0.5% Triton X-100 for 10 min. The cell lysate was collected and serially diluted, plated on LB agar medium, and cultured overnight at 37 °C for colony formation, and the bacterial colony-forming units (CFUs) was quantified. The bacterial amount was displayed in a square bacterial culture dish accordingly. The bacterial count measured at 2 h post-infection represents the total bacterial load both intracellularly and adhering extracellularly. The CFU count obtained after gentamicin treatment reflects the surviving intracellular bacterial load. The survival capacity of bacteria within host cells was assessed by comparing CFU counts at different time points relative to the initial 2-h infection. All experiments were independently replicated three times to ensure result reliability.

### Animal experiments

To investigate the effects of OMVs and OMV-induced ERS on APEC infection in vivo, a systemic infection experiment using a chick model was conducted to assess bacterial colonization in the trachea, lungs, liver, and spleen tissues of chicks [25, 26]. The experimental design included the following groups: Control, APEC, APECΔ*ypjA*, 4-PBA, and APEC + 4-PBA. Seven-day-old Roman chicks were randomly assigned to groups of ten each. Sample size was determined on the basis of previous research experience and preliminary experiments to achieve the required statistical power for detecting the anticipated effect (power ≥ 0.8, significance level *α* = 0.05). Chicks in each group were infected via tracheal injection with 1 × 10^9^ CFU per chick. Starting from the second day post-infection, 4-PBA (50 mg/kg) was continuously administered intraperitoneally to the 4-PBA and APEC + 4-PBA groups, while PBS was administered to the control group. On days 3 and 6, chicks were euthanized, and tissues were collected for pathological examination. Tissue samples were homogenized in PBS (1 mL/0.1 g) using a homogenizer, serially diluted, and spread onto LB agar plates to determine bacterial counts.

Additionally, portions of tissue samples (trachea, left lung, left hepatic lobe, and spleen) were immediately fixed in 4% paraformaldehyde, embedded in paraffin, sectioned, dewaxed, and stained with hematoxylin and eosin (HE). Portions of the lung and spleen were subjected to immunohistochemical staining using a specific antibody against GRP78/BiP. Histopathological lesions were evaluated using a semiquantitative 0–4 scoring system, where 0 = no lesion, 1 = minimal, 2 = mild, 3 = moderate, and 4 = severe. This scoring system was adapted from refs. [27, 28], with appropriate modifications based on the characteristics of the present experimental model. Pathological observations and scoring were performed by independent pathologists who were blinded to the group assignment information.

### Statistical analysis

All data are presented as the mean ± standard error of the mean (SEM) of multiple independent samples, with results derived from at least three independent replicate experiments. Statistical analyses were performed using Student’s *t*-test, one-way or two-way analysis of variance (ANOVA) with two-tailed tests, and controlling for family error rates, and effect sizes with 95% confidence intervals are reported where applicable. All statistical analyses were performed using GraphPad Prism 8 software. Statistical significance is indicated according to the following criteria: *p < 0.05, **p < 0.01, ***p < 0.001.

## Results

### APEC leads to systemic infection by secreting OMVs to evade macrophage immune clearance

**Figure 1:**
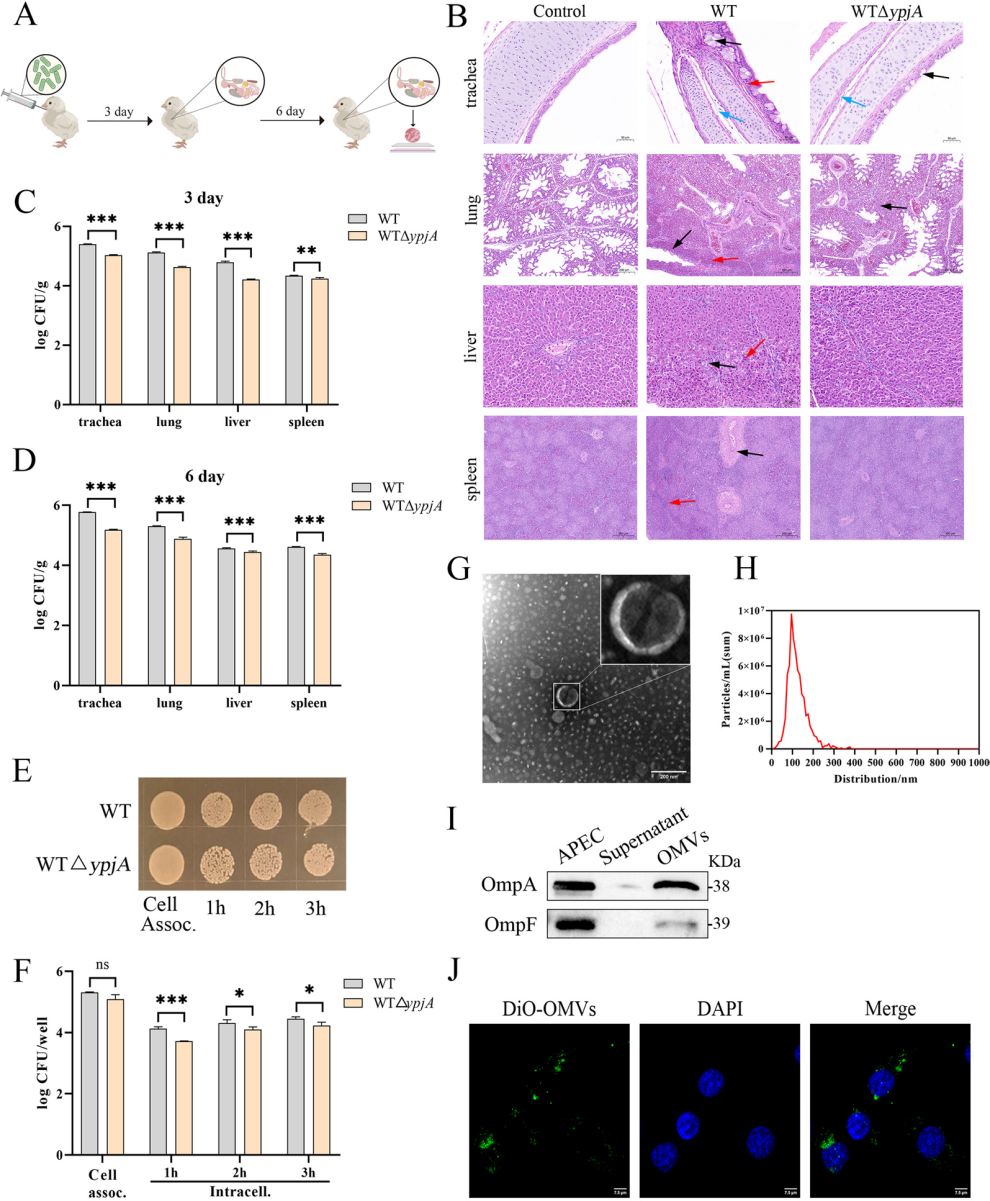
**APEC leads to systemic infection by secreting OMVs to evade macrophage immune clearance**. **A** Flowchart of chick infection. **B** Hematoxylin and eosin (HE) staining of the trachea, lung, liver, and spleen tissues from chicks infected with WT or WTΔ*ypjA* (1 × 10^9^ CFU/chick) on day 6 post-infection. Trachea: degeneration and hyperplasia of epithelial mucosal cells (black arrow), diffuse neutrophilic infiltration (red arrow), tracheal wall with mucus and inflammatory exudate (blue arrow); lung: widened pulmonary septa (black arrow), inflammatory cell infiltration in the interstitium (red arrow); liver: hepatocytes show lytic degeneration (black arrow) with inflammatory cell infiltration (red arrow); spleen: hyperplasia of lymphoid follicles (black arrow), blurred demarcation between white and red pulp (red arrow). Scale bar, 50 µm or 200 µm. **C**, **D** Bacterial loads in trachea, lungs, liver, and spleen at day 3 (**C**) and day 6 (**D**) post-infection. **C** (95% CI): trachea: [0.31–0.42]; lungs: [0.43–0.54]; liver: [0.53–0.64]; spleen: [0.04–0.16]. **D** (95% CI): trachea: [0.52–0.65]; lungs: [0.36–0.49]; liver: [0.06–0.19]; spleen: [0.19–0.32]. **E**, **F** Intracellular survival of WT and WTΔ*ypjA* after infection of HD11 cells. Square culture plates represent 1/100 bacterial load (*n* = 3). **G**, **H** Analysis of OMV morphology and concentration. Scale bar, 200 nm. **I** Western blot analysis of OmpA and OmpF proteins in APEC lysates, supernatant, and OMVs. **J** Confocal imaging of DiO-labeled OMVs entering HD11 cells; no signal in dye control. Representative of three experiments. Scale bar, 7.5 μm. *n* represents three biological replicates; data points indicate independent cultures. Bar charts show mean ± standard error of the mean (SEM) following two-way ANOVA with Sidak correction (*p < 0.05, **p < 0.01, ***p < 0.001). Effect sizes with 95% CIs are reported in panels (**C**) and (**D**).

To assess the role of OMVs in the infection process in vivo, we established three experimental groups: a control group, a wild-type APEC infection group (WT), and an OMV secretion-deficient strain infection group (WTΔ*ypjA*). Tissue samples from the trachea, lungs, liver, and spleen of chicks were collected at different time points for histopathological examination and bacterial load detection (Figure 1A). The histopathological results revealed that the tracheal epithelial mucosal cells in the WT group showed obvious degeneration and hyperplasia, with diffuse infiltration of neutrophils in the lamina propria and mucus and inflammatory exudates in the tracheal wall mucosa. In contrast, the tracheal epithelial mucosal cells in the WTΔ*ypjA* group showed slight degeneration, with trace amounts of mucus in the tracheal wall mucosa. The lung tissue in the WT group exhibited significantly widened interlobular septa, reduced alveolar airspace, and inflammatory cell infiltration within the pulmonary interstitium. In contrast, the WTΔ*ypjA* group showed only slight thickening of the pulmonary septa. The liver of the WT group showed a disordered arrangement of hepatocytes, with some hepatocytes undergoing hydropic degeneration and local inflammatory cell infiltration. In contrast, the WTΔ*ypjA* group exhibited only a disordered arrangement of hepatocytes without evident degeneration or inflammation. The spleen of the WT group showed marked hyperplasia of lymphoid follicles and an indistinct boundary between the white and red pulp. In contrast, the WTΔ*ypjA* group showed no obvious pathological changes (Figure 1B). To quantify the extent of pathological damage in each tissue, we further performed a histopathological scoring system to evaluate infection-related histopathological changes (Additional file 1A). The scoring data were in agreement with the microscopic observations, indicating more severe pathological damage in the WT group than in the WTΔ*ypjA* group. Bacterial load detection results indicated that bacterial load in all tissues increased gradually with infection time, but compared with the WT group, the bacterial load in the WTΔ*ypjA* group was significantly reduced in all tissues (Figures 1C, D). In addition, in an in vitro infection experiment using HD11 cells as a model, the intracellular survival rate of the WT group strain was higher than that of the WTΔ*ypjA* group (Figures 1E, F).

We isolated and purified OMVs secreted by APEC. Transmission electron microscopy revealed that OMVs exhibited a typical bilayered spherical structure (Figure 1G) with particle sizes mainly ranging from 20 to 400 nm (Figure 1H), consistent with the typical characteristics of OMVs. Western blot analysis confirmed the presence of OMV protein markers OmpA and OmpF [29]. The results showed that both bacterial lysates and OMVs contained OmpA and OmpF, while the culture supernatant showed relatively low levels of these proteins (Figure 1I), further confirming that the isolated particles were indeed OMVs. Additionally, OMVs were labeled with the fluorescent dye DiO to visualize their internalization. Laser confocal microscopy clearly captured distinct green fluorescence signals in HD11 cells, predominantly distributed within the cytoplasm (Figure 1J).

### APEC OMVs induce ERS in HD11 cells to evade immune clearance by macrophages

**Figure 2:**
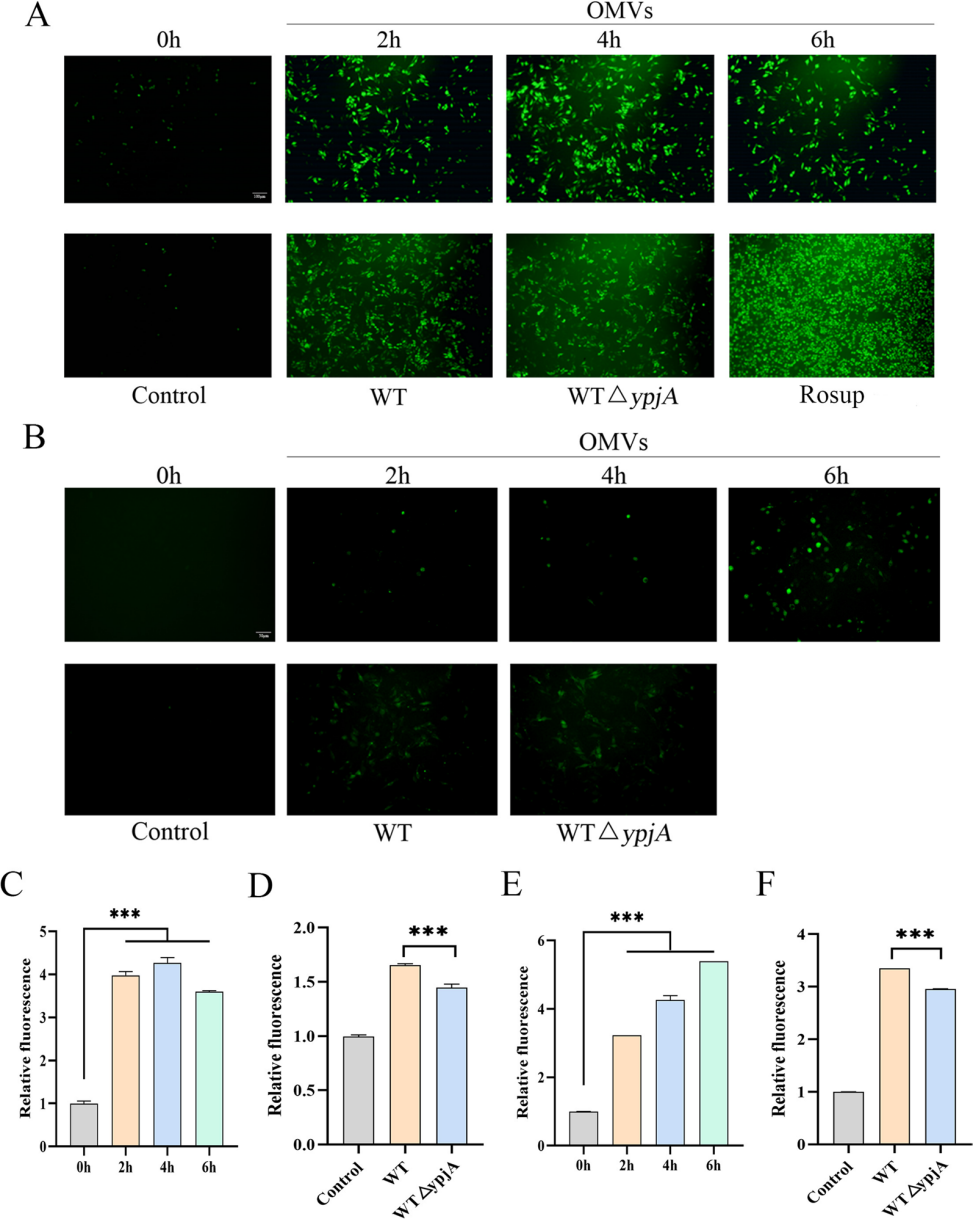
**APEC OMVs induce ROS accumulation and Ca**^**2**^**⁺ release in HD11 cells**. **A** HD11 cells were infected with WT or WTΔ*ypjA* at MOI = 100 for 2 h. HD11 cells were treated with OMVs (100 µg/mL) for 0–6 h, and intracellular ROS levels were measured. Data represent one of three independent experiments. Scale bar, 100 µm. **B** HD11 cells were infected with WT and WTΔ*ypjA* at MOI = 100 for 2 h. HD11 cells were treated with OMVs (100 µg/mL) or 0–6 h, and intracellular Ca^2+^ levels were measured. Data represent one of three independent experiments. Scale bar, 50 µm. **C**, **D** Quantitative analysis of fluorescence images (**A**) using ImageJ software. **E**, **F** Quantitative analysis of fluorescence images (**B**) using ImageJ software. Data points represent independent cultures. Bar charts display mean ± standard error of the mean (SEM). Analysis was performed using Student’s *t*-test. (***p < 0.001).

Given the critical roles of ROS and Ca^2+^ in regulating macrophage function and infection-related signaling pathways, we first evaluated their temporal changes during the early stage of infection. Accordingly, HD11 cells were treated with WT, WTΔ*ypjA*, or OMVs, and the intracellular levels of ROS and Ca^2+^ were measured (Figures 2A, B). The results showed that, with prolonged OMV treatment, the levels of ROS and Ca^2+^ in HD11 cells increased significantly, and the fluorescence intensity induced by the WT group was significantly higher than that of the WTΔ*ypjA* group (Figures 2C–F).

**Figure 3:**
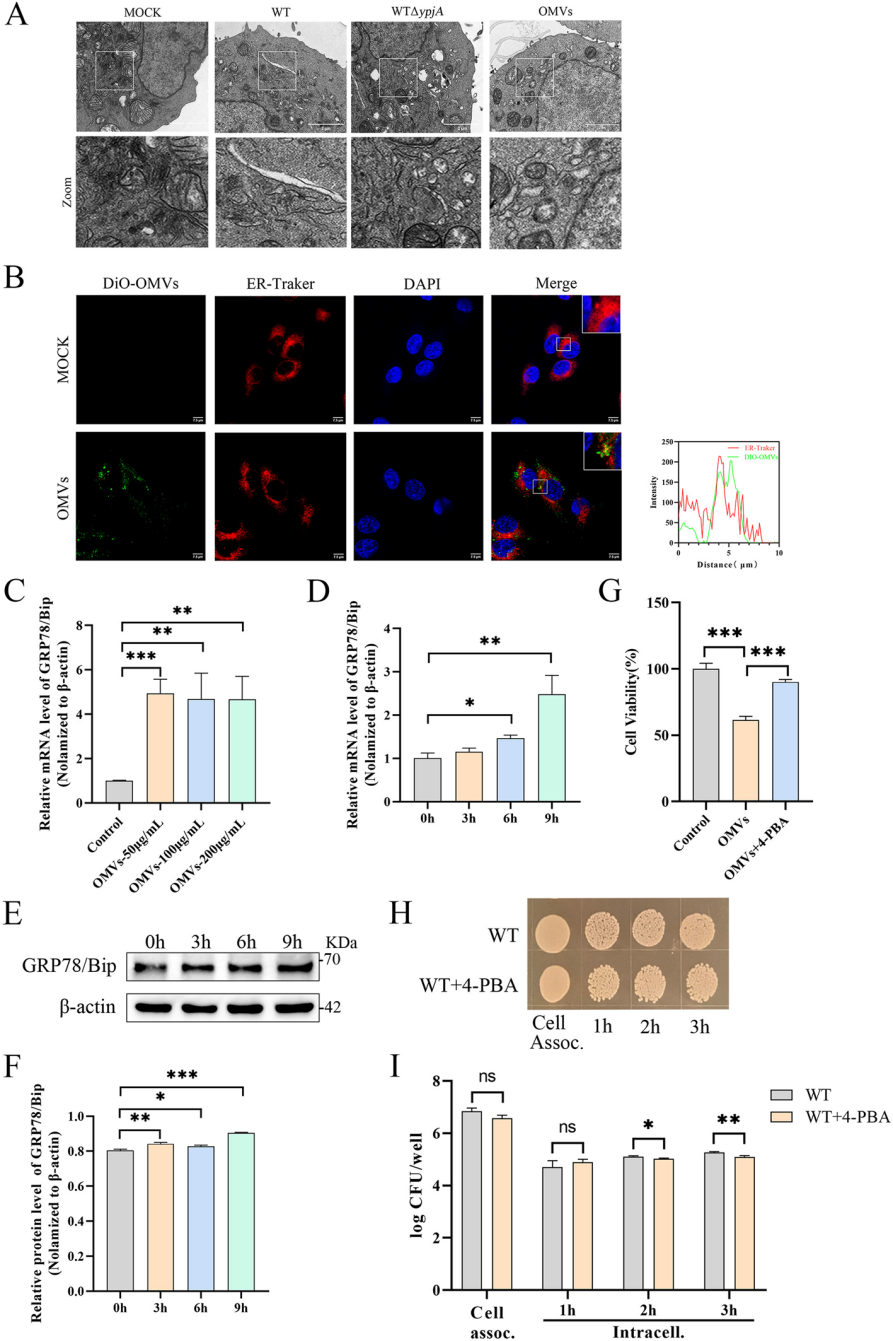
**APEC OMVs induce ERS in HD11 cells to evade immune clearance by macrophages**. Transmission electron microscopy shows ER network expansion and swelling in HD11 cells treated with WT, WTΔ*ypjA*, or OMVs. The data represent one of three independent experiments. Scale bar, 2 μm. **B** OMVs colocalized with ER. DiO-OMVs were incubated with HD11 cells at 37 °C for 6 h, the ER was labeled with ER-Tracker Red, and laser confocal microscopy imaging was performed. The data represent one of three independent experiments. Scale bar, 7.5 μm. Colocalization scatter plot was generated using ImageJ software and GraphPad Prism 8. **C** HD11 cells were treated with 50–200 µg/mL OMVs for 6 h, and the relative mRNA level of *GRP78/BiP* was determined by qPCR. (*n* = 3) **D** HD11 cells were treated with OMVs (100 µg/mL) for 0–9 h, and the relative mRNA level of *GRP78/BiP* was determined by qPCR. (*n* = 3) **E**, **F** HD11 cells were treated with OMVs (100 µg/mL) for 0–9 h, and western blot was used to detect the expression of GRP78/BiP protein in cell lysates and gray value analysis was performed using ImageJ software (**F**). (*n* = 3) **G** Cell viability of HD11 cells treated with OMVs (200 µg/mL) for 12 h was detected by CCK-8 in the absence or presence of 4-PBA (2 mM). (*n* = 3) **H**, **I** Intracellular survival of HD11 cells infected with WT at MOI = 100 in the absence or presence of 4-PBA (2 mM). Square culture plates showed 1/ 100 of the bacterial load. (*n* = 3). *n* represents three biological replicates; data points indicate independent culture systems. Bar charts display mean ± standard error of the mean (SEM). Data were analyzed using Student’s *t*-test and two-way ANOVA with Sidak correction. (*p < 0.05, **p < 0.01, ***p < 0.001).

We observed the ultrathin sections of HD11 cells treated with WT, WTΔ*ypjA*, and OMVs by transmission electron microscopy. The results showed that, compared with the mock group, the ER network appeared expanded and swollen in the three infected groups, and compared with the WT group, the degree of expansion was reduced in the WTΔ*ypjA* group, indicating that OMVs can alter the ultrastructure of the ER (Figure 3A). Laser confocal microscopy revealed that OMVs co-localized with the ER of HD11 cells (Figure 3B). Subsequently, we examined the transcription and protein levels of the marker protein GRP78/BiP of ERS by qPCR and western blot. The results showed that the relative messenger RNA (mRNA) level of *GRP78/BiP* was significantly upregulated in a concentration- and time-dependent manner following OMV treatment (Figures 3C, D), and the protein level also increased in a time-dependent manner (Figures 3E, F). Compared with the WT group, WTΔ*ypjA* infection resulted in reduced GRP78/BiP protein level (Additional file 2A). Furthermore, treatment with ROS scavenger Mito-TEMPO also significantly decreased the GRP78/BiP protein level (Additional file 2B).

To investigate whether OMV-induced ERS is involved in the survival of APEC in host macrophages, we pretreated HD11 cells with the ERS inhibitor 4-PBA. The results showed that 4-PBA significantly increased the viability of HD11 cells (Figure 3G) and reduced the intracellular survival of APEC in HD11 cells (Figures 3H, I).

### APEC OMVs activate UPR signaling to induce ERS

**Figure 4:**
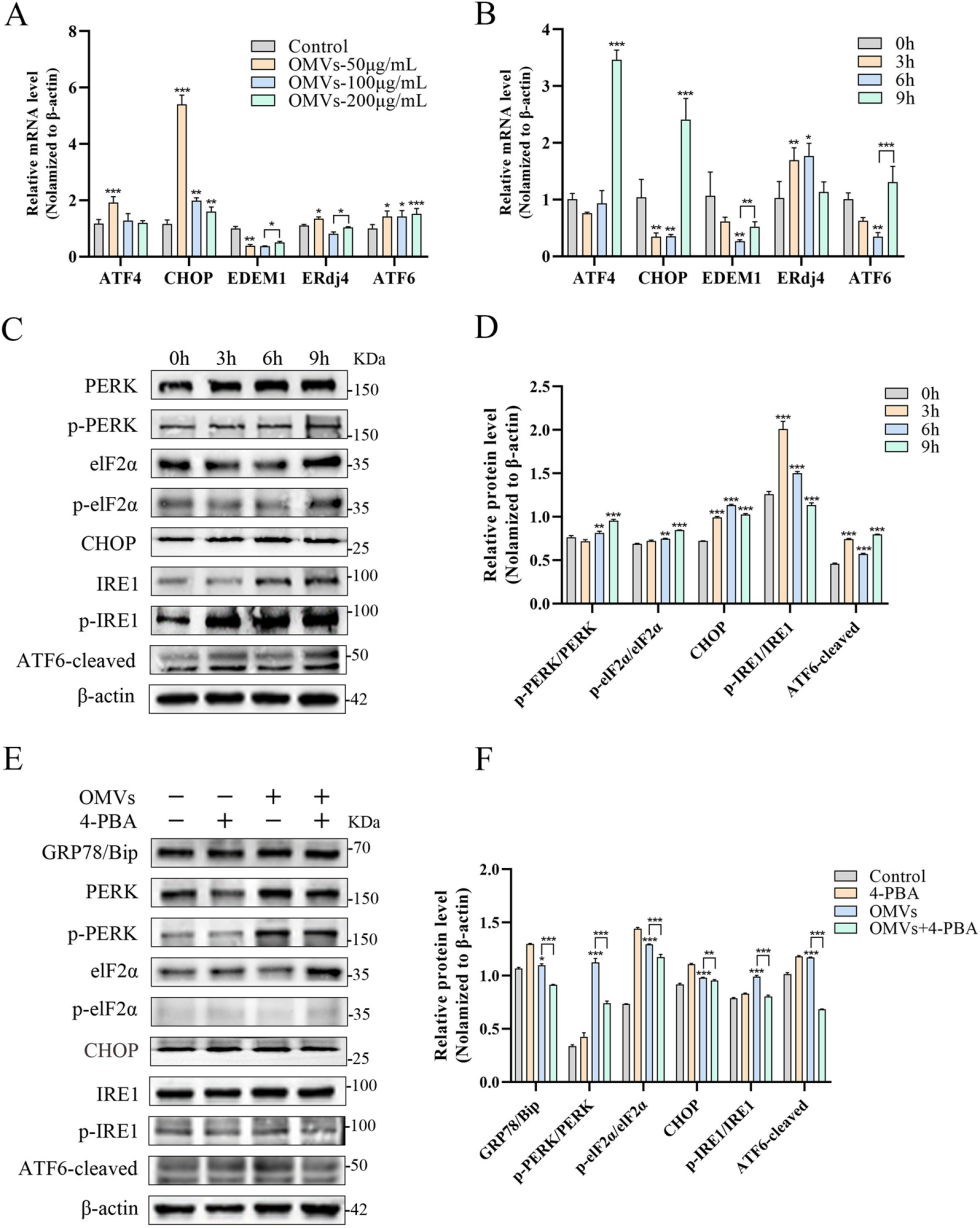
**APEC OMVs activate UPR signaling to induce ERS**. **A** HD11 cells were treated with 50–200 µg/mL OMVs for 6 h, and the relative mRNA levels of UPR downstream genes *ATF4*, *CHOP*, *EDEM1*, *ERdj4*, and *ATF6* were detected by qPCR. (*n* = 3) **B** HD11 cells were treated with OMVs (100 µg/mL) for 0–9 h, and qPCR was used to detect the relative mRNA levels of UPR downstream genes *ATF4*, *CHOP*, *EDEM1*, *ERdj4*, and *ATF6*. (*n* = 3) **C**, **D** HD11 cells were treated with OMVs (100 µg/mL) for 0–9 h, western blotting was used to detect the expression of PERK, p-PERK, eIF2α, p-eIF2α, CHOP, IRE1, p-IRE1, and ATF6 proteins in cell lysates, and gray value analysis was performed using ImageJ software (**D**). (*n* = 3). **E**, **F** HD11 cells were treated with OMVs (100 µg/mL) for 6 h in the absence or presence of 4-PBA (2 mM), western blot analysis was used to detect the expression of PERK, p-PERK, eIF2α, p-eIF2α, CHOP, IRE1, p-IRE1, and ATF6 proteins in cell lysates, and gray value analysis was performed using ImageJ software (**F**). (*n* = 3); *n* represents three biological replicates; data points indicate independent culture systems. Bar charts display mean ± standard error of the mean (SEM). Two-way ANOVA with Sidak correction was performed (*p < 0.05, **p < 0.01, ***p < 0.001).

To investigate the specific UPR pathways activated during OMV-induced ERS, we examined key molecules across three pathways using qPCR and western blot analysis. qPCR results revealed that PERK pathway-associated genes *ATF4* and *CHOP* were significantly upregulated with increasing OMV concentrations. *ERdj4*, an IRE1 pathway-associated gene, showed elevated expression across all treatment groups. *EDEM1* decreased at low concentrations but gradually recovered, while *ATF6* exhibited the highest expression in the high-concentration group (Figure 4A). As OMV treatment duration extended, *ATF4* peaked at 6 h, *CHOP* remained elevated between 6 and 9 h, *EDEM1* and *ERdj4* showed slight declines after rising between 3 and 6 h, while *ATF6* was significantly upregulated between 6 and 9 h (Figure 4B). These UPR-related genes displayed distinct time- and dose-dependent expression patterns. Western blot analysis further validated these changes (Figures 4C, D). With prolonged OMV treatment, the p-PERK/PERK protein ratio, p-eIF2α/eIF2α ratio, and levels of CHOP and cleaved ATF6 proteins progressively increased, while the p-IRE1/IRE1 ratio exhibited a dynamic pattern of initial elevation followed by decline. The 4-PBA treatment significantly reversed these protein expression trends (Figures 4E, F). Furthermore, reverse transcription (RT)-PCR detection of *XBP1* splicing revealed the presence of spliced *XBP1* mRNA variants in OMV-treated HD11 cells, which were absent in untreated cells, further confirming activation of the IRE1 pathway.

### APEC OMVs induce incomplete autophagy in HD11 cells

**Figure 5:**
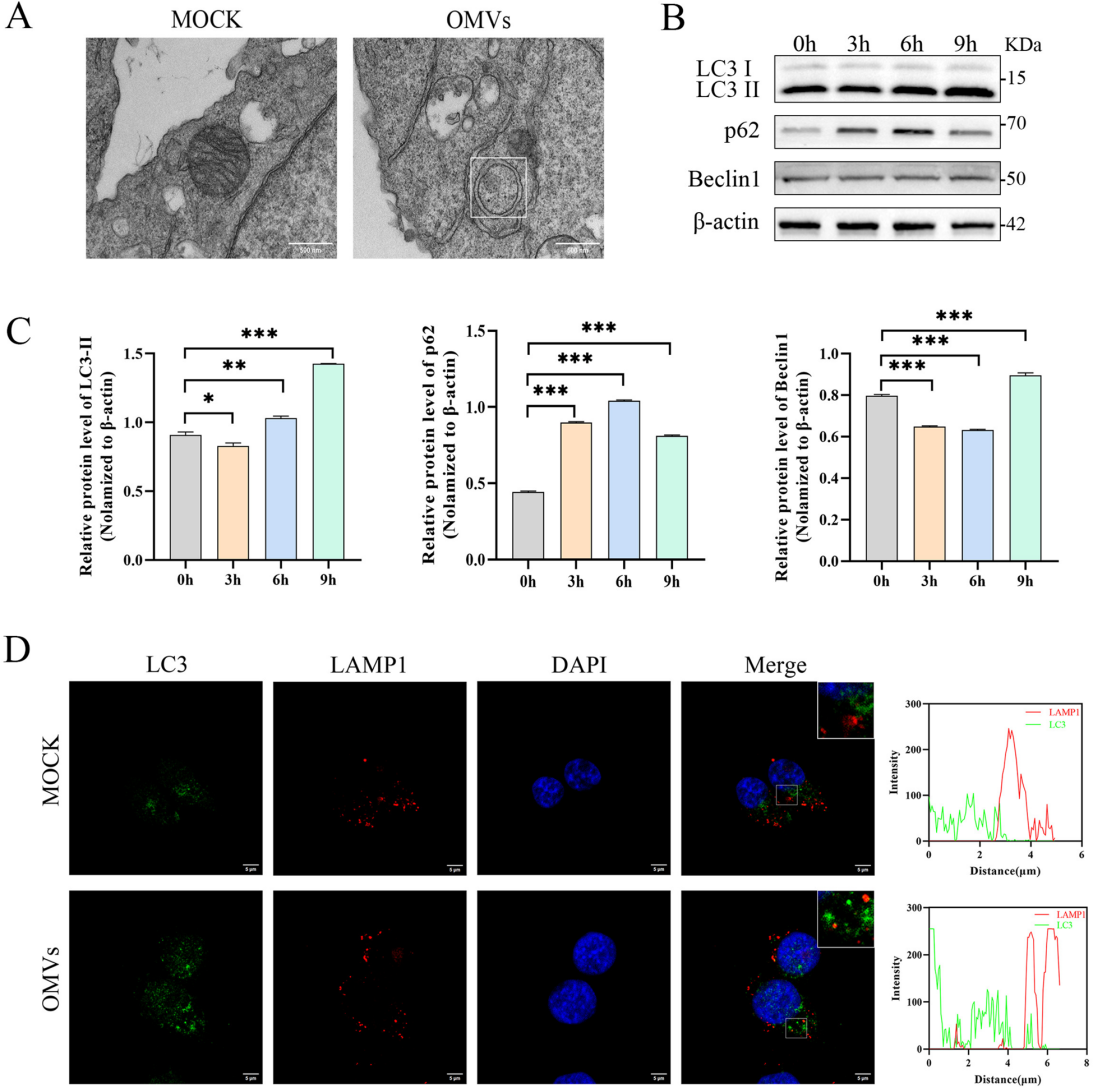
**APEC OMVs induce incomplete autophagy in HD11 cells**. **A** HD11 cells were treated with OMVs (100 µg/mL) for 6 h, and typical double-membrane autophagosomes were observed by transmission electron microscopy. Scale bar,500 nm. **B**, **C** HD11 cells were treated with OMVs (100 µg/mL) for 0–9 h, western blot analysis was performed to detect the expression of LC3-II, p62, and Beclin1 proteins in cell lysates, and gray value analysis was performed using ImageJ software (**C**) (*n* = 3). **D** Immunofluorescence was used to detect the effect of OMVs (100 µg/mL) on the colocalization of LC3 and the lysosomal marker protein LAMP1 in HD11 cells. The data are representative of three independent experiments. Scale bar, 5 μm. Colocalization scatter plots were generated using ImageJ software and GraphPad Prism 8. *n* represents three biological replicates, with each data point corresponding to an independent culture system. Bar charts display mean ± standard error of the mean (SEM), analyzed by Student’s -test (*p < 0.05, **p < 0.01, ***p < 0.001).

Transmission electron microscopy revealed the presence of typical double-membrane autophagosomes in HD11 cells treated with OMVs (Figure 5A), suggesting activation of autophagy. Moreover, western blot analysis revealed that infection with WTΔ*ypjA* significantly reduced LC3-II protein levels compared with the WT group (Additional file 2A). As the duration of OMV treatment increased, both LC3-II and p62 protein levels showed a significant increase, while Beclin1 protein levels initially decreased before increasing (Figures 5B, C). Furthermore, treatment with the ROS scavenger Mito-TEMPO markedly reduced LC3-II protein level (Additional file 2C).

Notably, p62, an autophagy substrate, was not degraded upon autophagy induction. Further confocal microscopy analysis revealed that LC3 exhibited a punctate fluorescence pattern but showed no apparent colocalization with the lysosomal marker LAMP1 (Figure 5D).

### APEC OMVs activate ERS to inhibit autophagosome degradation

**Figure 6:**
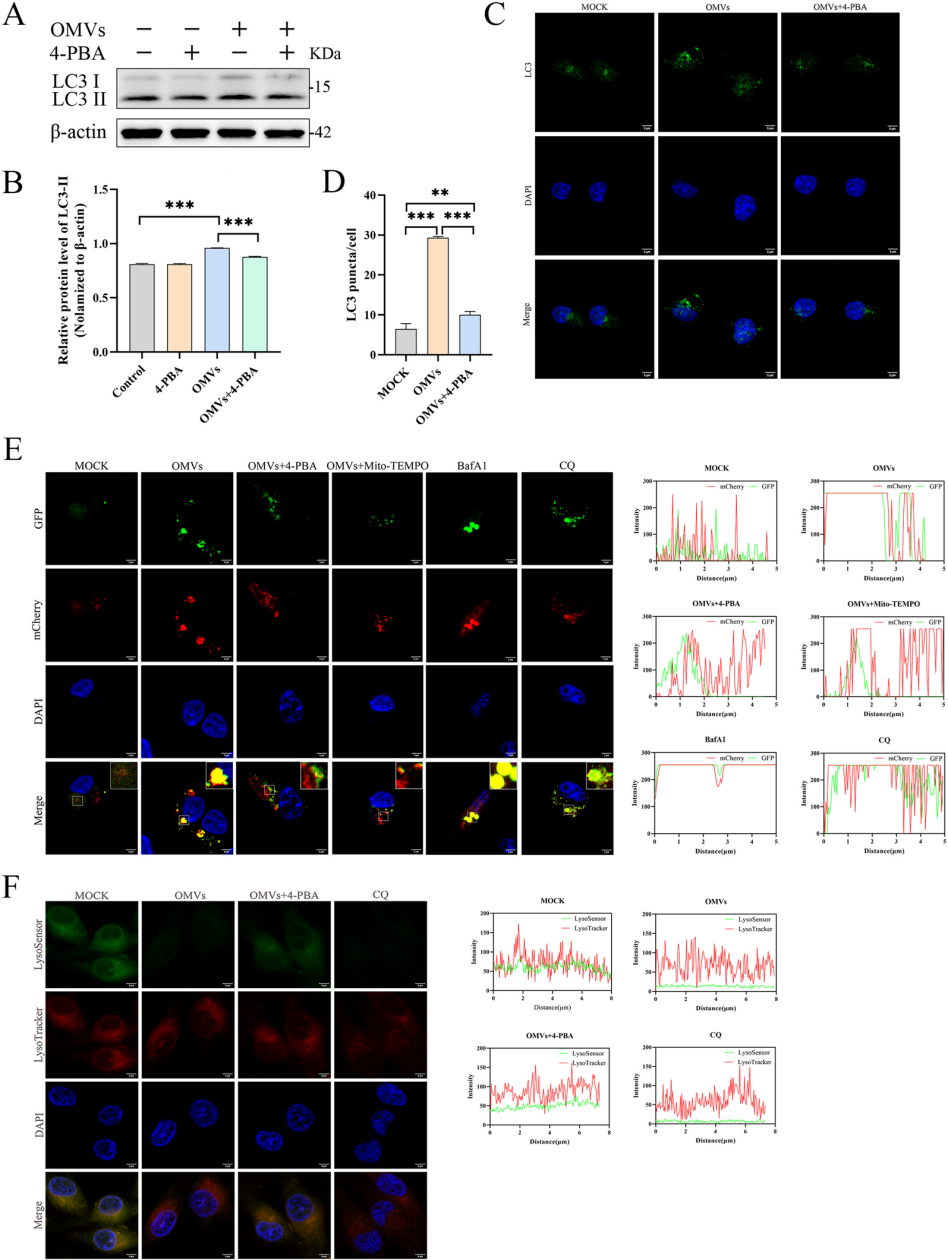
**APEC OMVs activate ERS to inhibit autophagosome degradation.** **A**, **B** HD11 cells were treated with OMVs (100 µg/mL) for 6 h in the absence or presence of 4-PBA (2 mM), western blot analysis was used to detect the expression of LC3-II protein in cell lysates, and gray value analysis was performed using ImageJ software (**B**) (*n* = 3). **C**, **D** HD11 cells were treated with OMVs (100 µg/mL) for 6 h in the absence or presence of 4-PBA (2 mM). LC3 dot aggregation was detected by immunofluorescence and statistically analyzed (**D**). Data represent one of three independent experiments. Scale bar, 5 µm. **E** HD11 cells transiently expressing the mCherry-GFP-LC3 plasmid were co-incubated with OMVs (100 µg/mL) for 6 h in the absence or presence of 4-PBA (2 mM) or Mito-TEMPO (10 µM), with CQ (10 µM) and bafA1 (100 nM) as controls. Fluorescence intensity of mCherry and GFP was detected in cells using laser confocal microscopy. Data represent one of three independent experiments; scale bar, 5 μm. Colocalization scatter plots were generated using ImageJ software and GraphPad Prism 8. **F** HD11 cells were treated with OMVs (100 µg/mL) for 6 h in the absence or presence of 4-PBA (2 mM), with CQ (10 µM) as a control. Fluorescence intensity of LysoSensor and LysoTracker in cells was detected by laser confocal microscopy. Data represent one of three independent experiments. Scale bar, 5 μm. Colocalization scatter plots were generated using ImageJ software and GraphPad Prism 8. represents three biological replicates; data points represent independent cultures. Bar charts show mean ± standard error of the mean (SEM); analysis performed using Student’s *t*-test (***p < 0.001).

We further explored whether autophagy induced by OMVs in HD11 cells depends on ERS. To this end, HD11 cells were pretreated with 4-PBA. Western blot results showed that 4-PBA treatment significantly reduced the expression level of LC3-II protein (Figures 6A, B). Meanwhile, confocal microscopy revealed a decrease in LC3 puncta aggregation (Figures 6C, D). These findings further indicate that, when ERS was alleviated by 4-PBA, autophagy levels decreased accordingly.

To further investigate whether OMVs inhibit autophagosome degradation by regulating ERS, we used an mCherry-GFP-LC3 dual-fluorescent tandem sensor to detect autophagic flux and lysosomal acidification. The results showed that, compared with the mock group, both GFP and mCherry signals were markedly enhanced in the OMV-treated group, indicating impairment of autophagic flux and suppression of lysosomal acidification. This phenomenon resembled the effects observed with the lysosomal acidification inhibitors chloroquine (CQ) and bafA1. Pretreatment with 4-PBA or Mito-TEMPO partially reversed the OMV-induced increase in GFP fluorescence (Figure 6E).

To further validate whether OMV-induced ERS affects the lysosomal acidification process, we employed the lysosome-specific fluorescent probes LysoTracker and LysoSensor to assess. The results showed that LysoSensor fluorescence intensity was markedly reduced following OMV treatment, exhibiting effects similar to those observed with CQ treatment. However, following 4-PBA pretreatment, the LysoSensor fluorescence intensity was significantly enhanced compared with the OMV-treated group (Figure 6F).

### APEC OMVs induce ERS to promote APEC systemic infection

**Figure 7:**
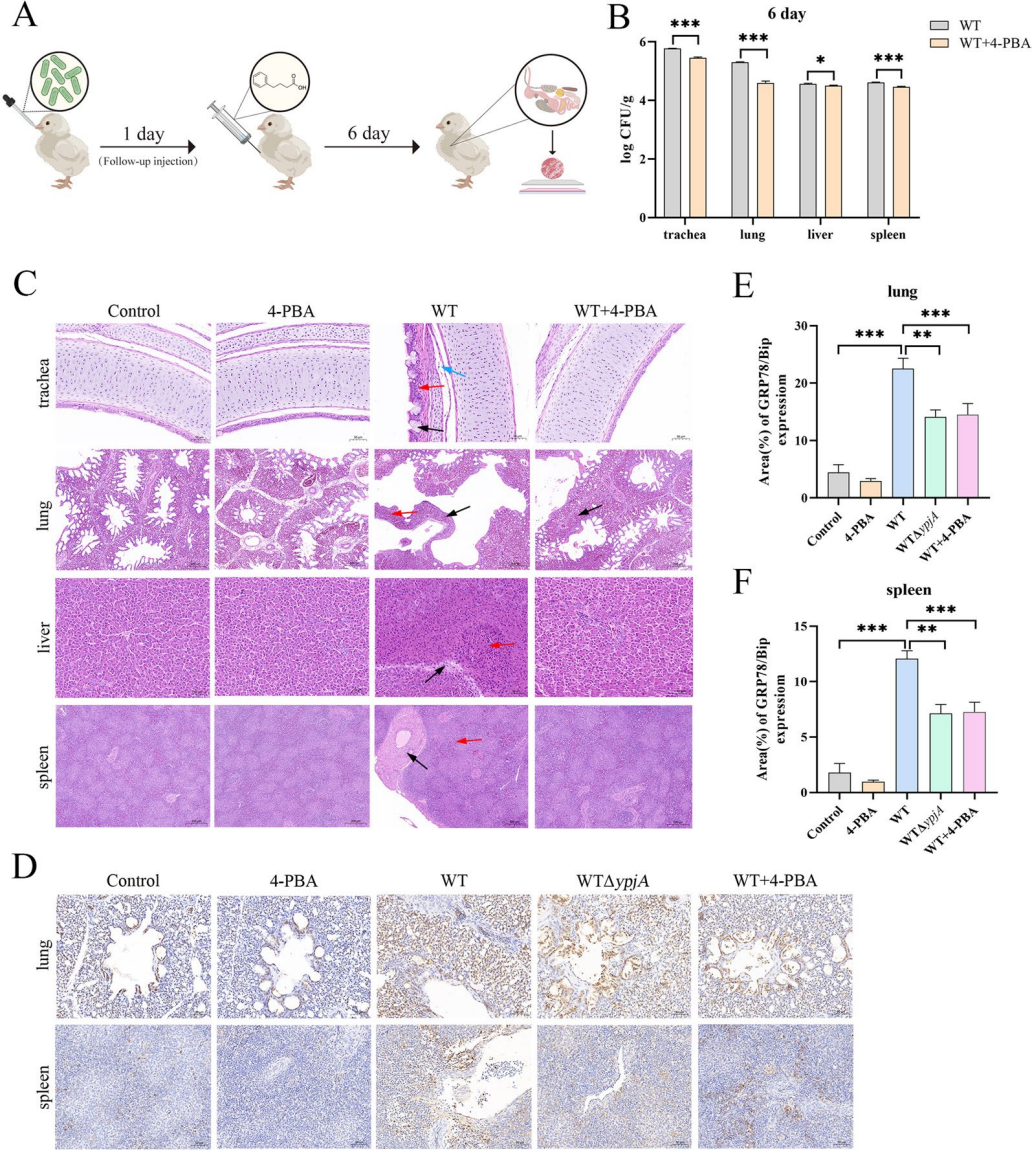
**APEC OMVs induce ERS to promote APEC systemic infection**. Flowchart of the infection experiment using chicks treated with 4-PBA. **B** On the sixth day after WT infection of chicks (1 × 10 CFU/chick), the number of bacteria (CFU) was counted in the trachea, lungs, liver, and spleen tissues of the chicks in the absence or presence of 4-PBA (50 mg/kg). Trachea: 95% CI [0.26–0.38]; lungs: 95% CI [0.65–0.78]; liver: 95% CI [0.004–0.13]; spleen: 95% CI [0.08–0.21]. **C** On the sixth day after WT infection of chicks (1 × 10^9^ CFU/chick), HE staining was performed on tracheal, lung, liver, and spleen tissues of the chicks in the absence or presence of 4-PBA (50 mg/kg). Trachea: degeneration and hyperplasia of epithelial mucosal cells (black arrow), diffuse neutrophilic infiltration (red arrow), mucus and inflammatory exudate in the mucosa of the tracheal wall (blue arrow); lung: markedly widened pulmonary septa (black arrow), inflammatory cell infiltration in the pulmonary interstitium (red arrow); liver: hepatocytes show lytic degeneration (black arrow) with inflammatory cell infiltration (red arrow); spleen: marked hyperplasia of lymphoid follicles (black arrow), blurred demarcation between white and red pulp (red arrow). Scale bar, 50 µm or 200 µm. **D**–**F** GRP78/BiP immunofluorescence staining was performed on lung and spleen tissues from chicks on day 6 post-infection (**D**) and analyzed using ImageJ software (**E**, **F**). Scale bar, 50 µm. Data points represent independent cultures; bar charts show mean ± standard error of the mean (SEM). Two-way ANOVA with Sidak correction was performed (*p < 0.05, **p < 0.01, ***p < 0.001). Effect sizes with 95% confidence intervals are reported in panel (**B**).

We infected chicks with APEC and began daily 4-PBA administration the following day to modulate ERS levels. Six days after infection, pathological observations and bacterial load detection were performed on the trachea, lungs, liver, and spleen of the chicks (Figure 7A). Bacterial load analysis revealed that, compared with the WT group alone, 4-PBA treatment markedly reduced the bacterial load in all examined tissues (Figure 7B). Histopathological examination showed that lesions in the WT group were consistent with those described previously (Figure 1B). Following continuous 4-PBA administration, tissue damage was alleviated: pulmonary interstitial septa showed only mild thickening, the liver showed slight disorganization of hepatic cords, and no obvious pathological changes were observed in the trachea or spleen (Figure 7C). Histopathological scoring results were consistent with the aforementioned pathological observations (Additional file 1B). Additionally, we detected the levels of the ERS marker protein GRP78/BiP in lung and spleen tissues by immunohistochemical staining (Figure 7D). The results showed that, compared with the mock group, the level of the ERS marker GRP78/BiP in lung and spleen tissues was significantly increased in the WT group; however, compared with the WT group, the level of GRP78/BiP was significantly decreased in both the WTΔ*ypjA* group and WT + 4-PBA group (Figures 7E, F).

## Discussion

The successful establishment of infection by pathogens depends on their key capabilities, including adhesion, invasion, and intracellular survival within the host. Increasing evidence suggests that APEC is not merely an opportunistic pathogen but can invade the host and establish infection through multiple routes, such as the respiratory tract, skin wounds, or the birth canal, even in the absence of obvious exogenous triggers [30, 31]. Previous studies [32] and this study have both confirmed that APEC can effectively colonize multiple tissues such as the trachea, lungs, liver, and spleen, and the severity of systemic infection progressively increases with prolonged infection. Recent studies using a mouse systemic infection model further demonstrated that, compared with commensal *Escherichia coli*, extraintestinal pathogenic *Escherichia coli* (ExPEC) exhibited significantly slower clearance rates in the liver and spleen [33], suggesting that this type of pathogen possesses the ability to persist long-term within the host, thereby delaying infection resolution. Although this study did not systematically describe the spread and clearance kinetics of APEC in systemic tissues, the existing results still indicate that it is necessary to conduct further quantitative research using the chick infection model to comprehensively analyze the replication dynamics, persistence, clearance, and intertissue transmission patterns of APEC within the host.

After breaching the host barrier, APEC must effectively evade host immune clearance to establish a persistent infection [34]. In this study, the APEC strain AE81 isolated from the lung tissue of a chick that died from *Escherichia coli* sepsis was found to survive and replicate within macrophages. Notably, compared with neutrophil cells, macrophages possess stronger bactericidal capacity; therefore, APEC must employ multiple strategies to evade phagocytic uptake and killing, including avoiding recognition and phagocytosis, inhibiting phagosome–lysosome fusion, escaping from phagosomes, tolerating the antimicrobial environment within phagolysosomes, or evading autophagy recognition [35, 36]. OMVs as virulence factor delivery vectors showed consistent function in *Salmonella* [8], enteropathogenic *Escherichia coli* (ExPEC) [37], and the APEC AE81 strain in this study: OMV secretion-deficient strains exhibit reduced survival capacity within macrophages, milder histopathological lesions in infected chicks, and significantly lower bacterial loads, indicating that OMVs play a critical role in promoting systemic infection and intracellular survival. Similar phenomena have been observed in a mouse infection model of *Mycobacterium tuberculosis*: OMV treatment significantly enhanced bacterial replication in the lungs and accelerates its spread to the spleen [38]. These findings collectively suggest that the absence of OMV formation capacity limits the release of APEC virulence factors, thereby weakening its overall pathogenicity. Therefore, OMVs are not only core elements for APEC to achieve immune evasion and intracellular survival but also key determinants for maintaining and enhancing its pathogenicity.

The UPR is an important protective mechanism for restoring cellular homeostasis under ERS, and the ER is also involved in activating innate immune signals against pathogen invasion [14]. Numerous studies have shown that viral infections often trigger the UPR, which is related to the high dependence of viruses on host protein synthesis mechanisms; some viruses can even actively regulate the UPR to optimize their protein expression and replication, and maintaining host cell survival [39]. Recent studies have revealed that the UPR also plays multiple roles during bacterial infections. This study provides direct evidence that APEC activates all three branches of the UPR in HD11 cells via OMVs, indicating that OMV-mediated persistent ERS plays a crucial role in the APEC infection process. Further experiments showed that pretreatment of HD11 cells with the ERS inhibitor 4-PBA significantly reduced the intracellular survival of APEC. In the chick infection model, 4-PBA treatment also led to a decrease in tissue bacterial load and a reduction in pathological damage, confirming that ERS induced by OMVs exerts an infection-promoting effect. In this process, OMVs act as signal carriers, entering host cells via endocytosis, releasing “cargo” such as proteins, nucleic acids, metabolites, and toxins [40], and regulating host cell signal transduction and the functions of important cell organelles such as the ER and lysosomes, thereby disrupting cellular homeostasis and suppressing immune responses. However, how microbial pathogens utilize ER-related signaling pathways and membrane transport mechanisms to achieve immune evasion remains to be further explored.

Autophagy is a key metabolic process that maintains intracellular homeostasis. It involves the formation of autophagosomes, which are double-membrane structures that engulf cytoplasmic contents, including invading pathogens, and are subsequently degraded after fusion with lysosomes [41]. However, certain pathogens have evolved diverse strategies to counteract or evade autophagy; For example, *Shigella flexneri* delivers the effector protein IcsB through its type III secretion system (T3SS), which inhibits the host’s autophagic recognition of intracellular bacteria [42]. This indicates that, although autophagy serves as an important anti-infective defense mechanism, it can still be actively evaded by pathogens. In this study, transmission electron microscopy revealed typical double-membrane autophagosomes in HD11 cells treated with OMVs, but APEC was not effectively cleared. Further analysis revealed the absence of colocalization between the autophagy marker protein LC3 and the lysosomal marker LAMP1, indicating that the autophagy flux was blocked at the autophagosome–lysosome fusion stage, leading to incomplete autophagy. Additionally, OMVs disrupted lysosomal acidification, leading to abnormal accumulation of autophagosomes and consequently impairing the bactericidal capacity of macrophages. These findings are highly consistent with those of David et al. [43] who reported that ExPEC-derived OMVs impeded autophagosome–lysosome fusion and acidification processes, further supporting the central role of OMVs in bacterial evasion of autophagy.

Numerous studies have shown that ROS and Ca^2+^ not only participate in the induction and regulation of ERS but also are key signaling molecules in the autophagy process [44, 45]. On the one hand, ERS is often accompanied by disruption of ER calcium homeostasis and enhanced oxidative stress. Excessive ROS and cytosolic Ca^2+^ can amplify stress responses via the UPR pathway, impairing protein folding and organelle function. On the other hand, elevated Ca^2+^ levels can promote ROS generation, while ROS can act as upstream signals to directly or indirectly activate the autophagy initiation complex, driving autophagosome formation [46]. Studies have shown that the lytic toxin LLO produced by *Listeria monocytogenes* disrupts intracellular Ca^2+^ homeostasis through pore formation, activates all three UPR pathways, and induces ERS-mediated apoptosis, although the exact mechanism remains unclear [47]. In this study, the ERS inhibitor 4-PBA was applied as an intervention. It was found that 4-PBA not only significantly alleviated OMV-induced ERS but also partially restored autophagosome–lysosome fusion and lysosomal acidification function, indicating that ERS may facilitate the formation of incomplete autophagy by inhibiting the degradative phase of autophagy, thereby validating our previous hypothesis of cross-regulation between the ER and autophagy degradation. Considering previous studies showing that pathogens such as *Mycobacterium tuberculosis* [48] and *Staphylococcus aureus* [49] can utilize host autophagy mechanisms to enhance intracellular survival and replication, we speculate that OMV-induced ERS and lysosomal dysfunction synergistically promote APEC immune evasion. Although 4-PBA effectively alleviates ERS as a chemical chaperone, it may also influence other cellular pathways, such as inflammatory responses and apoptosis [50, 51]. Therefore, future studies will further validate this by testing orthogonal ERS modulators such as tauroursodeoxycholic acid (TUDCA) or employing genetic interference (e.g., CHOP knockdown). Moreover, the specific components of OMVs that induce ERS remain unclear. OMVs are rich in lipopolysaccharides (LPS), microbial nucleic acids, cyclic dinucleotides such as c-di-adenosine monophosphate (c-di-AMP) and cyclic di-guanosine monophosphate (c-di-GMP), and various toxins [52], which may individually or synergistically trigger and amplify the ERS response. Future studies should employ systematic screening and validation experiments to clarify the role of key virulence factors in OMVs within this mechanism. Furthermore, this study explores only one OMV-mediated mechanism in APEC infection. However, APEC infection likely involves multiple interacting mechanisms that may synergistically exacerbate the infection process [53]. Hence, future research should expand experimental model to comprehensively investigate other secretory systems of APEC, virulence factors, and host immune responses, thereby fully elucidating the complexity of APEC infection and its immune evasion strategies.

**Figure 8:**
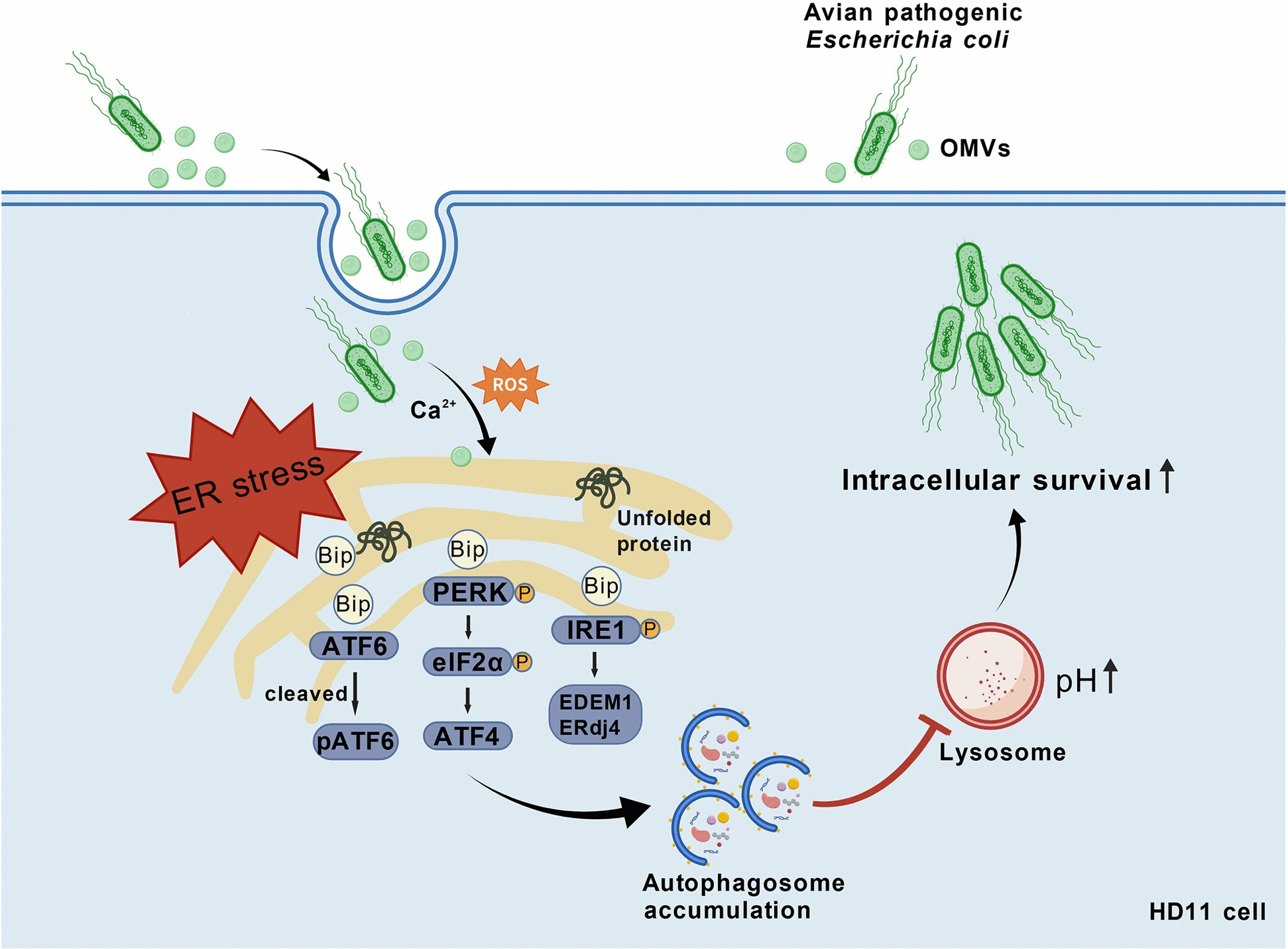
**OMVs secreted by avian pathogenic *Escherichia coli* promote its survival within macrophages and systemic infection by inducing ERS-mediated autophagy flux blockade**. APEC-secreted OMVs, upon uptake by HD11 cells, induce ROS accumulation and Ca^2+^ release, triggering ERS and activating UPR pathways, including the PERK, IRE1, and ATF6 signaling branches, leading to the expression of stress-related factors such as GRP78/BiP and CHOP. The sustained activation of ERS inhibits autophagosome degradation and disrupts the acidic environment of lysosomes, thereby preventing autophagosomes from fusing with lysosomes and impairing the phagocytic clearance capacity of macrophages. Collectively, these abnormal conditions facilitate APEC survival within HD11 cells and enable immune evasion, ultimately promoting bacterial dissemination and systemic infection in the host.

In summary, the results of this study demonstrate that OMVs secreted by APEC comprehensively activate the UPR signaling pathway by inducing Ca^2+^ homeostasis imbalance and ROS accumulation in HD11 cells, thereby triggering persistent ERS. Concurrently, OMVs inhibit autophagosome degradation and lysosomal acidification, ultimately suppressing the host’s clearance function and promoting systemic infection by APEC (Figure 8). This study provides a novel perspective on the role of OMVs in the pathogenic mechanism of APEC and lays a theoretical foundation for further exploration of immune intervention strategies against avian colibacillosis.

## Supplementary Information

**Additional file 1: Histopathological scores of trachea, lung, liver, and spleen in chicks infected with APEC. **Histopathological changes were scored on a scale from 0 to 4 (0 = no lesion; 4 = extremely severe lesion). Data are presented as mean ± SD (*n* = 5 per group). Statistical significance was determined by two-way ANOVA with Sidak correction (**p <0.01, ***p <0.001).

**Additional file 2: Effect of reduced APEC-secreted OMVs and ROS clearance on ERS and autophagy.** (A) WT or WTΔ*ypjA* infected HD11 cells for 4 h with MOI = 100. Western blot analysis was performed to detect GRP78/BiP and LC3-II expression in cell lysates, and gray value analysis was performed using ImageJ software (*n* = 3). (B, C) HD11 cells were treated with OMVs (100 µg/mL) for 6 h in the absence or presence of Mito-TEMPO (10 μM). GRP78/BiP and LC3-II protein expression in cell lysates was detected by western blot and gray value analysis was performed using ImageJ software (*n* = 3). *n* represents three biological replicates; data points indicate independent cultures; bar charts show mean ± standard error of the mean (SEM). Statistical analysis was performed using Student’s *t*-test and one-way ANOVA (**p < 0.01, ***p < 0.001).

**Additional file 3: *XBP1* cleavage assay.** The specific endonuclease activity of IRE1 cleaves a 26-nt intron containing a *PstI* restriction site within the precursor mRNA of the transcription factor *XBP1*. Cleavage generates the active transcription factor XBP1s, which induces expression of downstream target genes, whereas the unprocessed form XBP1u, inhibits their expression (A–C). RT-PCR amplification was performed using XBP1-specific primers. The PCR products were digested with *PstI* and visualized on a 1.5% agarose gel, with fragment sizes as shown in (D).

## Data Availability

No datasets were generated or analyzed during the current study.
