# Supplementary figures and images for: Outer membrane vesicles secreted by avian pathogenic Escherichia coli promote its survival within macrophages and systemic infection by inducing endoplasmic reticulum stress-mediated autophagy flux blockade
Source: Vet Res. 2026 Jan 27;57:34. doi: 10.1186/s13567-025-01679-6 (PMC12918585; doi:10.1186/s13567-025-01679-6)

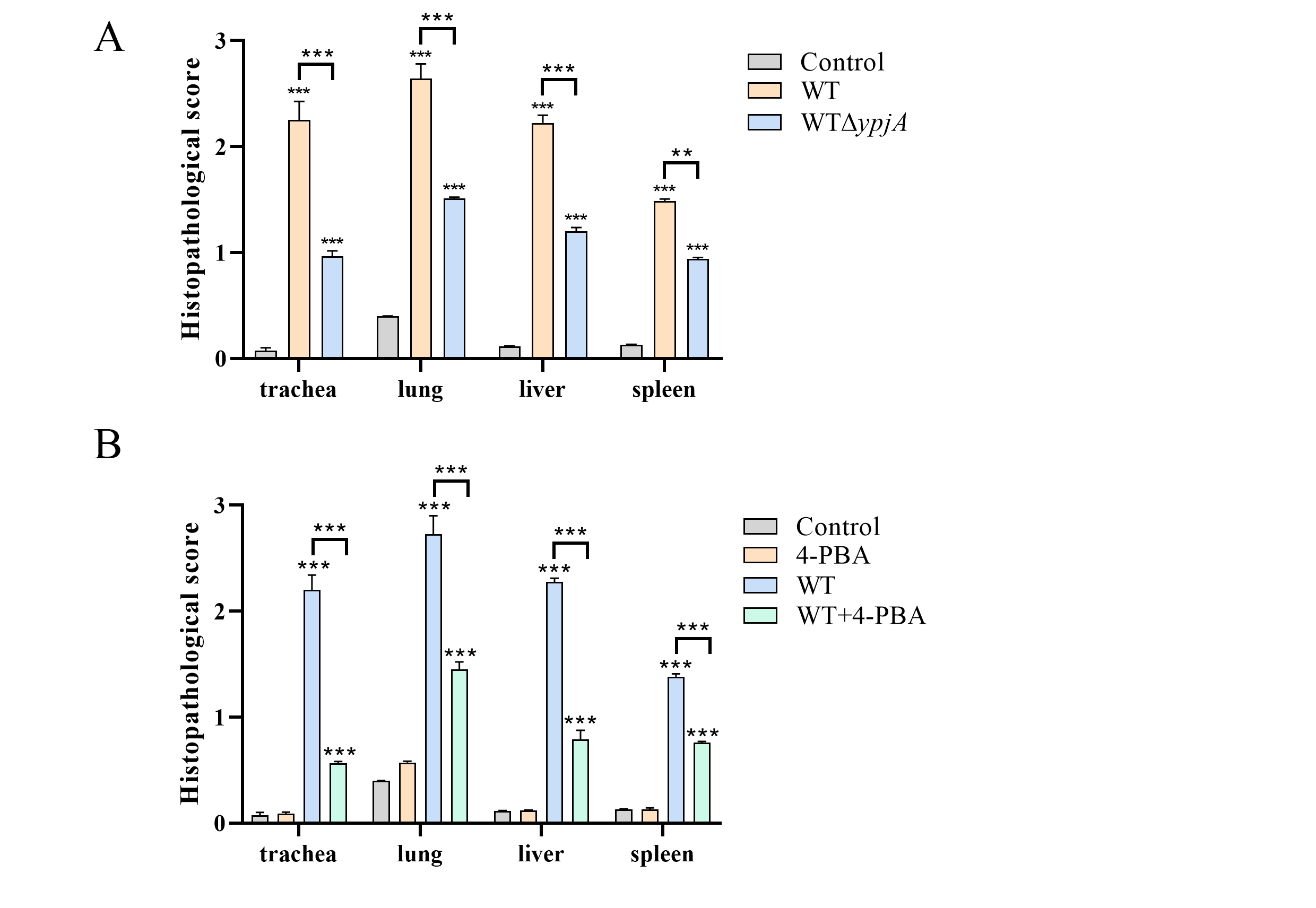

Supplement: Supplementary file 1 — Additional file 1: Histopathological scores of trachea, lung, liver, and spleen in chicks infected with APEC. Histopathological changes were scored on a scale from 0 to 4 (0 = no lesion; 4 = extremely severe lesion). Data are presented as mean ± SD (n = 5 per group). Statistical significance was determined by two-way ANOVA with Sidak correction (**p <0.01, ***p <0.001). [file 13567_2025_1679_MOESM1_ESM.tif]

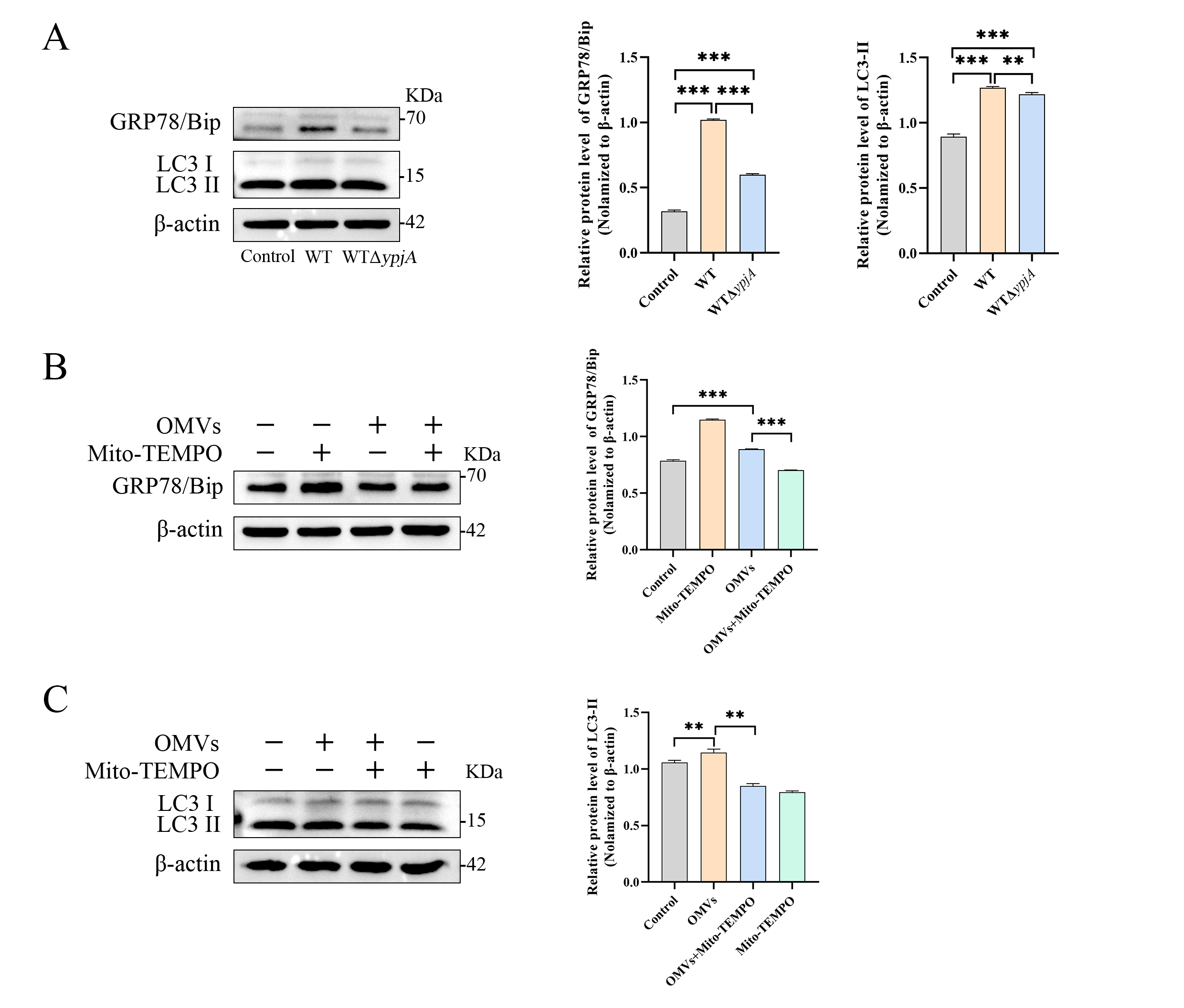

Supplement: Supplementary file 2 — Additional file 2: Effect of reduced APEC-secreted OMVs and ROS clearance on ERS and autophagy. (A) WT or WTΔypjA infected HD11 cells for 4 h with MOI = 100. Western blot analysis was performed to detect GRP78/BiP and LC3-II expression in cell lysates, and gray value analysis was performed using ImageJ software (n = 3). (B, C) HD11 cells were treated with OMVs (100 µg/mL) for 6 h in the absence or presence of Mito-TEMPO (10 μM). GRP78/BiP and LC3-II protein expression in cell lysates was detected by western blot and gray value analysis was performed using ImageJ software (n = 3). n represents three biological replicates; data points indicate independent cultures; bar charts show mean ± standard error of the mean (SEM). Statistical analysis was performed using Student’s t-test and one-way ANOVA (**p < 0.01, ***p < 0.001). [file 13567_2025_1679_MOESM2_ESM.tif]

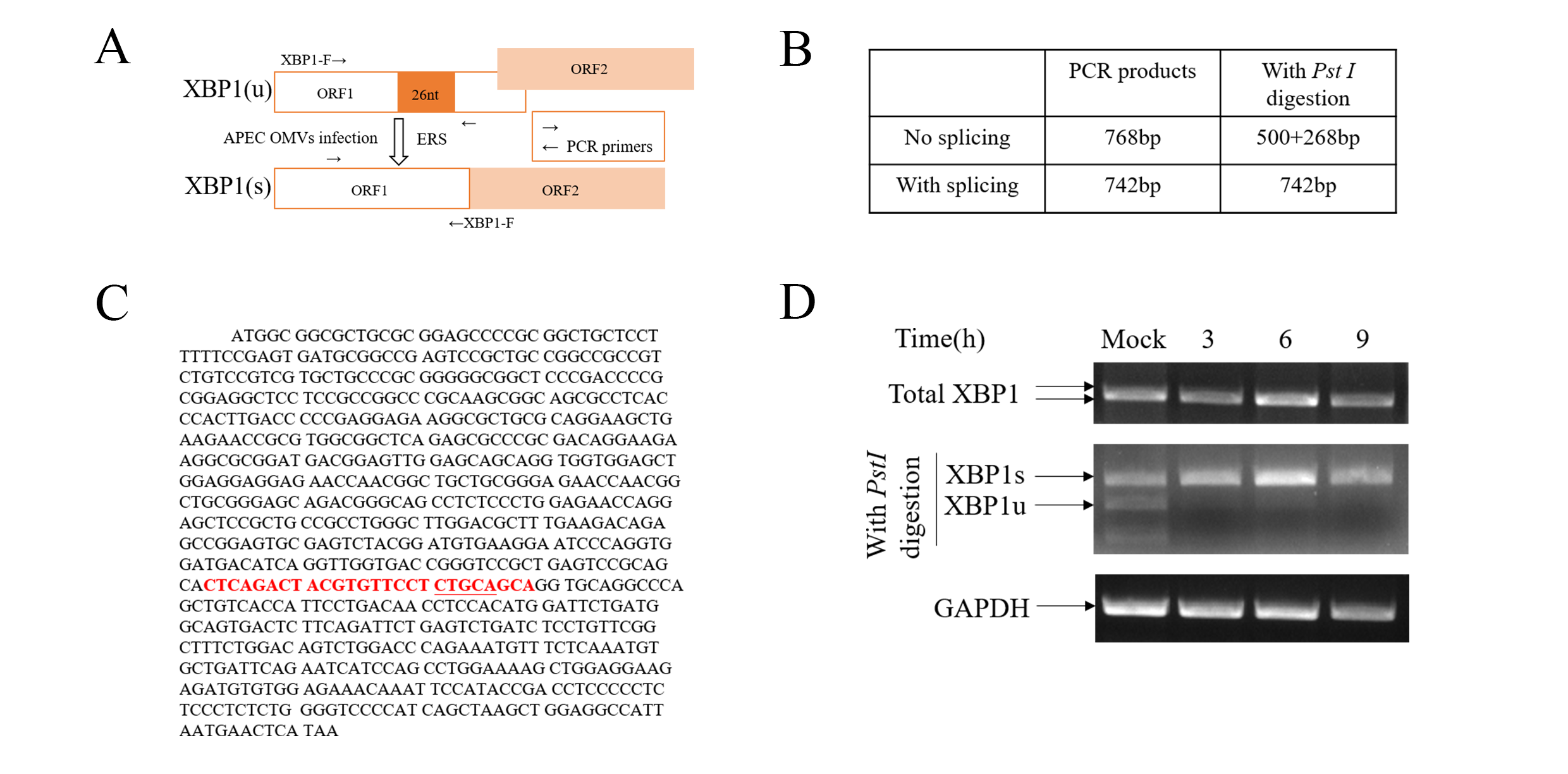

Supplement: Supplementary file 3 — Additional file 3: XBP1 cleavage assay. The specific endonuclease activity of IRE1 cleaves a 26-nt intron containing a PstI restriction site within the precursor mRNA of the transcription factor XBP1. Cleavage generates the active transcription factor XBP1s, which induces expression of downstream target genes, whereas the unprocessed form XBP1u, inhibits their expression (A–C). RT-PCR amplification was performed using XBP1-specific primers. The PCR products were digested with PstI and visualized on a 1.5% agarose gel, with fragment sizes as shown in (D). [file 13567_2025_1679_MOESM3_ESM.tif]
